# A Machine Learning Algorithm Suggests Repurposing Opportunities for Targeting Selected GPCRs

**DOI:** 10.3390/ijms251810230

**Published:** 2024-09-23

**Authors:** Shayma El-Atawneh, Amiram Goldblum

**Affiliations:** Molecular Modelling and Drug Design Lab, Institute for Drug Research and Fraunhofer Project Center for Drug Discovery and Delivery, Faculty of Medicine, The Hebrew University of Jerusalem, Jerusalem 9112001, Israel; shayma.el-atawneh@mail.huji.ac.il

**Keywords:** repurposing, GPCRs, ISE, machine learning, drug discovery, cannabinoid 2 receptors, histamine receptors, dopamine receptors

## Abstract

Repurposing utilizes existing drugs with known safety profiles and discovers new uses by combining experimental and computational approaches. The integration of computational methods has greatly advanced drug repurposing, offering a rational approach and reducing the risk of failure in these efforts. Recognizing the potential for drug repurposing, we employed our Iterative Stochastic Elimination (ISE) algorithm to screen known drugs from the DrugBank database. Repurposing in our hands is based on computer models of the actions of ligands: the ISE algorithm is a machine learning tool that creates ligand-based models by distinguishing between the physicochemical properties of known drugs and those of decoys. The models are large sets of “filters” made out, each, of molecular properties. We screen and score external sets of molecules (in our case- the DrugBank molecules) by our agonism and antagonism models based on published data (i.e., IC_50_, K_i_, or EC_50_) and pick the top-scoring molecules as candidates for experiments. Such agonist and antagonist models for six G-protein coupled receptors (GPCRs) families facilitated the identification of repurposing opportunities. Our screening revealed 5982 new potential molecular actions (agonists, antagonists), which suggest repurposing candidates for the cannabinoid 2 (CB2), histamine (H1, H3, and H4), and dopamine 3 (D3) receptors, which may be useful to treat conditions such as neuroinflammation, obesity, allergic dermatitis, and drug abuse. These sets of best candidates should now be examined by experimentalists: based on previous such experiments, there is a very high chance of discovering novel highly bioactive molecules.

## 1. Introduction

Drugs typically interact with around six different biological targets, based on an analysis of 4767 interactions involving 802 drugs and 480 targets from various databases [1]. The G protein-coupled receptor (GPCR) network, with only 22% of all targets tested, was found to have 2646 (56% of all interactions) between 396 drugs (~50%) and 106 targets [1]. That result indicates the importance of repurposing among the GPCRs. Furthermore, a study focusing on FDA-approved drugs (2689 different targets interacting with 2186 FDA-approved) revealed an extensive drug-target network with a huge component containing 4376 interactions, involving targets primarily related to metabolism, cardiovascular diseases, and cancer, among others [2]. Illicit drugs demonstrate a similar pattern, targeting an average of 6.6 human genes each. These drugs primarily fall into four categories: depressants, stimulants, analgesics, and steroids [3]. These findings highlight the complex and multifaceted nature of drug action, often involving multiple targets, a phenomenon known as polypharmacology.

Polypharmacology analysis could identify potential drug repurposing (or repositioning) [4,5], offering significant benefits to the pharmaceutical industry by circumventing the costly and lengthy pharmacokinetic and toxicological profiling tests. Repurposing can shorten the drug development timeline from 10–17 years to just 3–12 years [6]. Additionally, the cost of bringing a repurposed drug to market is estimated to be around USD 300 million, compared to approximately USD 2–3 billion for bringing a new chemical entity to the therapeutics market [6].

Drugs withdrawn from the market due to severe adverse effects or lack of efficacy can be repurposed for other therapeutic indications. Most were discovered serendipitously, such as Miltefosine, Sildenafil, and Thalidomide [4,5,6]. In a recent review, Corbett et al. [7] described compounds collected from various studies exploring their potential repositioning to treat Alzheimer’s disease (AD). Remarkably, these compounds originated from different pathologies and therapeutic applications, such as type 2 diabetes mellitus, hypertension, and antibiotics. Repurposing can also provide a means to develop treatments for neglected diseases that might not otherwise be profitable for pharmaceutical companies [8].

Computational methods can prioritize the drug discovery process more efficiently (faster and reducing costs), thus substantially impacting drug development despite the inherent complexity of the underlying polypharmacology. Current computational methods for drug discovery and repurposing encompass a variety of approaches, including disease-centric, target-centric, and drug-centric strategies, with virtual screening (VS) being the most used in silico tool to search for repurposing opportunities so far [9]. In silico methods enable to set relationships between different types of data to create new information and knowledge that enhances pattern recognition and predictive capabilities through machine learning tools like deep neural networks [10,11]. Additionally, such algorithms have been highlighted in drug repurposing for diseases like COVID-19, emphasizing the re-evaluation of existing drugs and the biological and computational interpretation of AI-guided repurposing [12]. The Computational Analysis of Novel Drug Opportunities (CANDO) [13] platform used docking and drug-protein interaction analysis on a proteomic scale to predict potential molecular interactions that could lead to novel pharmacotherapeutics [14]. DrugRep [15] performs receptor-based (by identifying possible binding pockets) and ligand-similarity-based virtual screening to find new targets for existing drugs.

Van Noort et al. [16] used drug-induced gene-expression profile similarity to retrieve novel candidate drugs for colorectal cancer. Through computational analysis, they identified three compounds: citalopram (antidepressant), troglitazone (antidiabetic), and enilconazole (fungicide), as potential treatments. Experimental validations for the anticancer activities of all three compounds using in vitro tests and subcutaneous tumor models in mice confirmed their efficacy in inhibiting tumor growth, proliferation, and migration. Notably, citalopram demonstrated significant anti-tumor activity in a preclinical model, reducing tumor size and metastasis [16].

Hongbo et al. used docking and gene expression data mining to identify potential drug candidates for AD [17]. By docking 1553 FDA-approved drugs onto seven major AD drug targets, 211 approved drugs showed high binding free energies for all seven targets. Then, gene expression profiles for 74 drugs (those with corresponding gene expression profiles) were used to verify the docking results. Seven representative repositioned drugs were tested for their protective effects on Aβ25–35 aggregation cytotoxicity. The results identified risperidone, droperidol, glimepiride, and glipizide as potential multi-target candidates for the treatment of AD.

A study [18] that compares the performance of different web services based on chemical similarity assessment (ChemProt, SuperPred, SEA, SwissTargetPrediction, and TargetHunter) and machine learning methods (ChemProt and PASS) to predict original and new therapeutic indications found that machine learning-based methods generally outperform chemical similarity-based methods, especially for predicting novel repurposed indications.

Here, we use our GPCR models for agonism or antagonism (AGANT) [19] as a screening platform to propose new repurposing opportunities for known drugs from the DrugBank database [20]. We focus on a subset of receptors: cannabinoid 2 (CB2), histamine (H1, H3, and H4), and dopamine 3 (D3) receptors, suggesting potential repurposing candidates for various conditions like neuroinflammation, obesity, and allergic dermatitis. The AGANT models for 31 GPCRs were built using the “Iterative stochastic Elimination” (ISE) algorithm [21,22].

ISE offers solutions to extremely complex combinatorial problems [22]. In the case of drug discovery or repurposing, it produces models of many “filters”, each filter being a “solution” to the problem of distinguishing between known active molecules (e.g., IC_50_, K_i_, K_d_, K_a_, EC_50_) and inactive molecules, or between highly active molecules and weakly active ones—always on a single protein target. ISE requires variables, variable values, and a scoring method for all possible combinations of variable values. The ISE process begins by randomly sampling combinations of variables (we use only physicochemical properties of ligands, thereby avoiding structural similarity and increasing diversity) and evaluating their ability to distinguish between the “positives” (with known experimental data) and the “negatives” (no data). Statistical criteria are used (see Section 3) to determine which variables or variable values contribute consistently to bad filters that cannot distinguish between the two sets. Those variables are rejected, thus reducing the number of possible combinations. Identifying and eliminating consistently poor-performing values, ISE progressively narrows the search space in a few iterations, which comprise, each, a random construction of filters, screening of the whole learning set through each filter, and subsequent decisions on eliminating variables or variable values. This iterative process continues until a manageable number of combinations remain, allowing for a final exhaustive search of all remaining combinations and ranking the solutions by their efficiency. Each model is thus a set of filters (“solutions”) that differ in their efficiency and are scored (Section 3).

The model may subsequently score any molecule based on its ability to pass through the set of filters. Each filter score is added if a molecule passes it or is deducted if not. The total for each molecule makes it possible to compare it to other molecules. A higher score indicates a higher probability of the molecule being an agonist or antagonist. This scoring system aids in prioritizing molecules for further experimental evaluation. Large compound libraries can be efficiently filtered by setting a suitable score threshold, yielding a smaller, enriched set for subsequent analysis.

In many cases, screening through the ISE models is enough to produce a set of candidates for experimental testing. However, as these models are only based on properties, not on structural components, molecules of different sizes and flexibilities may have similar properties. We use docking after ligand-based modeling only to examine the chance of a molecule binding to a target and not to consider agonism or antagonism. Docking may be performed if the protein target structure is known—we use it to eliminate molecules that do not “fit” to the target despite their favorable score from our ligand-based modeling. Thus, the ISE ligand-based method is prioritized over the structure-based docking method.

## 2. Results and Discussion

### 2.1. Iterative Stochastic Elimination (ISE) Agonist/Antagonist Models

Based on our recent work [19], models for agonism and antagonism were built for 31 receptors from six families: Dopamine (D1–D5), 5-Hydroxytryptamine (5-HT), Muscarinic (M1–M5), Histamine (H1–H4), Opioid (Mu, Delta, and Kappa), and the cannabinoid (CB1 and CB2) receptors. The activity models were assessed using five-cross-validation and multiple metrics, including Matthews Correlation Coefficient (MCC) [23], Area Under the ROC Curve (AUC), True Positive Rate (TPR), and True Negative Rate (TNR) (Table S1, see also methods Section 3.1). The mean MCC for the agonist models ranges between 0.68–0.99 with an average of 0.84; for the antagonist models, it ranges between 0.55–0.95 and an average of 0.81 [19]. The AUC for agonist models ranges between 0.57–0.99. For the antagonist models, AUC ranges between 0.85–0.99 [19]. The TNR (specificity) is high for all models (>0.9), and the TPR or sensitivity is high for almost all models (53 models have a value above 0.5) (Table S1).

When evaluating our models, we acknowledge that imbalanced data, where molecules with AGANT data (positives) are significantly less than non-AGANT, can skew the interpretation of confusion matrix values. As seen in Table S1, our models achieve very high specificity (>0.94), but sensitivity varies across receptors. Reducing sensitivity might lead to missed opportunities, but we typically retain sufficient molecules for further in vitro testing. Thus, we utilize the MCC metric. MCC considers all confusion matrix components (see methods Section 3.1) and provides a more robust evaluation, especially for imbalanced learning sets like ours (1:100 positive-to-negative ratio) [24,25,26]. All our models have MCC values exceeding 0.5. Additionally, we consider AUC, which measures overall model performance across various classification thresholds. Most models in Table S1 have AUC values exceeding 0.8, reflecting efficient performance.

### 2.2. DrugBank Screening Results

#### 2.2.1. Drug-GPCR-Activity Matrix

The 7130 drugs of the DrugBank database were screened through the 59 AGANT models (except for three receptors: D5/5-HT1E/5-HT5A agonism due to lack of data). We compared our predictions to reported/ known AGANT. The 1212 reported activities between 361 drugs and 31 GPCRs were derived from three databases (DrugBank v. 5.1.9 [20], ChEMBL27 [27] and TTD [28]). The ISE models correctly predicted 56% of these 1212 AGANT and suggested a large number, 21,987, of new AGANT actions (molecules with any positive score, Table 1) [19]. To suggest new candidates, we choose a cutoff score with the highest true positives/false positives (TP/FP) rate (methods Section 3.1), meaning a higher chance to find true candidates and fewer false ones. We are interested in new unreported molecular actions, such as molecules that got a high score in our AGANT models but were not reported as AGANT.

#### 2.2.2. Drug-GPCR-Interaction Matrix

A drug that acts as an agonist or antagonist on a specific GPCR is interacting/connected to this receptor. The Drug-GPCRs-interaction matrix assigns “1” if there is a connection and assigns “0” if the drug has no action on that receptor (for both reported and predicted AGANT). The Drug-GPCRs-interaction matrix consists of 1165 reported connections between 361 drugs and the 31 GPCRs (reflecting the chance of binding to the receptor, as both agonists and antagonists must bind to affect the target). The success rate of predictions in this case (Table 2—out of 1165 reported interactions) is higher than that of AGANT predictions (out of the reported 1212). The receptors vary in performance (Table S2); the D2 model has 95% success above an index of zero, while the D1 model has only 16%.

**Figure 1:**
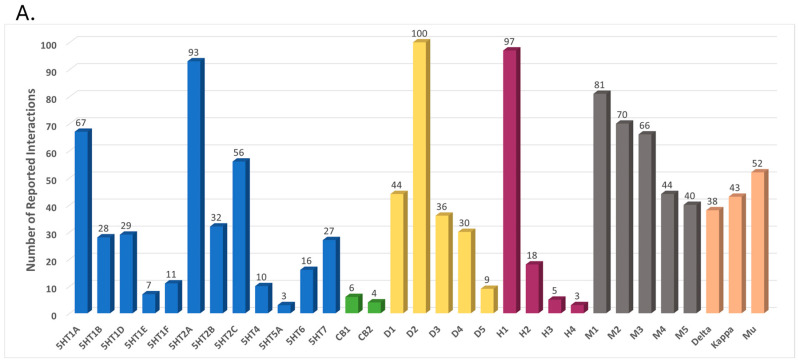

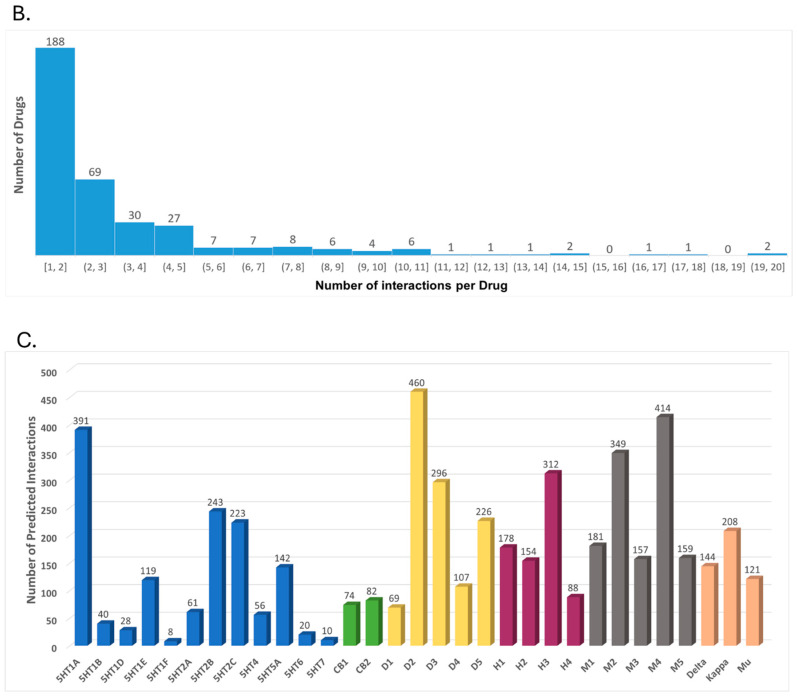
(**A**) Reported interactions for six GPCR families: 5-Hydroxytryptamine (serotonin, 5-HT)—blue, Cannabinoids—green, Dopamine (D1–5)—yellow, Histamine (H1–4)—pink, Muscarinic (M1–5)—gray and Opioid (Delta, Kappa, and Mu)—orange. (**B**) The number of reported interactions per drug. (**C**) Predicted interactions above a score of 0.7. 5120 interactions involving 1283 drugs interacting with the 31 GPCRs: 5-Hydroxytryptamine (serotonin, 5-HT)—blue, Cannabinoids—green, Dopamine (D1–5)—yellow, Histamine (H1–4)—pink, Muscarinic (M1–5)—gray and Opioid (Delta, Kappa, and Mu)—orange.

The minimum of three interactions (for 5-HT5A and histamine 4 (H4) receptor) raised to 100 interactions with the dopamine 2 (D2) receptor (Figure 1A). The 361 drugs have a range of 1–20 interactions and an average of 3.2 interactions per drug (Figure 1B). The majority (188 out of 361 drugs) have just one interaction with the selected GPCRs. Based on our previous experience in prediction analysis, we apply for all models a high score cutoff of 0.7 in order for the molecules to be further processed: 5120 new/unknown interactions were found, involving 1283 drugs interacting with the 31 GPCRs (Figure 1C). The correlation between the number of reported interactions and new predicted interactions per receptor is 0.43. We will focus on four predicted actions for further analysis: CB2R agonists (82 drugs), H3R antagonists (109 drugs), H1R/H4R antagonists (two drugs), and D3R antagonists (36 drugs). The structures for the four sets are provided in Figure S1.

### 2.3. Tanimoto Similarity

The sets with the reported data used to build each model are diverse, as well as the predicted drugs for each receptor (Table 3). Comparing the reported compounds to the predicted drugs for each receptor yields low average Tanimoto values (Table 3 and Figure S2).

### 2.4. Docking Results

Docking (a structure-based method) is used by us only following the screening using our ISE ligand-based method. The main purpose of docking in our hands is to eliminate molecules that have the correct physicochemical characteristics but do not fit the relevant binding sites well enough. To validate our docking method, we begin with re-docking, i.e., using a known crystal structure of a protein-ligand complex and docking the ligand using a computational method to compare it with the experiment. We shall use that docking method only if the docking finds a ligand position close to the experimental one, usually by measuring the Root Mean Square Distance (RMSD) between the docked and the experimental positions. We require that RMSD should be less than 3.0 Å.

The redocking results of the native ligands for each structure are listed in Table 4. All ligands fit well in the binding pocket with high docking scores (Figure S3A–D). The following paragraphs describe the redocking results in order to compare docking with experiment:

LEI-102 redocked to the CB2R with a docking score of −8.2 Kcal/mol. The H-bond with T114^3.33^ (Ballesteros–Weinstein numbering in superscript [29]) was retrieved, as well as the hydrophobic interactions with residues: F87^2.57^, S90^2.60^, F94^2.64^, F106^3.25^, K109^3.28^, I110^3.29^, F183^ECL2^, P184^ECL2^, I186^ECL2^, Y190^5.39^, L191^5.40^, W194^5.43^, M265^6.55^, F281^7.35^, and S285^7.39^ (Figure S4A).

The H3R antagonist (PF-03654746) has a high docking score of −12.8 Kcal/mol and two hydrogen bonds with D114^3.32^ and Y91^2.61^, which are shown to be essential for PF-03654746 activity [30]. Mutations in F193^ECL2^, Y374^6.51^, and E395^7.36^ can entirely abolish the PF-03654746 inhibition [30], and these interactions were retrieved in the docking process (Figure S4B).

For the third set (H1R/H4R antagonists), only H1R structure is available [31]. Doxepin, a first-generation antihistamine drug, interacts with highly conserved key residues W428^6.48^ (pi-pi stacking) and D107^3.32^ (salt bridge). D107^3.32^ has been shown to be essential for binding H1R antagonists and agonists in mutational studies [31]. Interactions with K191^5.39^ and/or K179^ECL2^ are part of the anion-binding region. Doxepin interacts only with K191^5.39^, in addition to other hydrophobic and polar residues (Figure S4C).

Eticlopride, the D3R antagonist, retrieved all 18 interactions reported in the X-ray structure [32], including the salt bridge with D110^3.32^ and interactions with the hydrophobic cavity formed by F345^6.51^ and F346^6.52^ in helix VI; V189^5.39^, S192^5.42^, and S193^5.43^ in helix V; V111^3.33^ in helix III, as well as I183^ECL2^ (Figure S4D). It got a higher RMSD value than the other redocked ligands. As shown in Figure S3D, the ethyl-pyrrolidine ring has a different orientation than the crystal structure ligand.

The docking results of the predicted drugs for each receptor are detailed in Tables S3–S6. In Table 5, the ranges of scores are listed for each receptor. Most of the drugs in CB2R were docked to the agonist binding site with high docking scores. However, only 49 of the predicted H3R drugs (out of 109) have a high docking score of less than −8 Kcal/mol compared to other H3R antagonists [30]. Of the two predicted drugs that interact with the H1R/H4R, only one got a high score of −10.5 Kcal/mol in the H1R structure. Seven of the last set of predicted D3 antagonists got a higher docking score than the native antagonist eticlopride (−7.9 Kcal/mol).

### 2.5. Repurposing Opportunities

To find repurposing opportunities, we search the 5982 new AGANT (above a score of 0.7). Here, we show a few examples of selected targets related to autoimmune and inflammation disorders, obesity, and central nervous system disorders (CNS). By picking drugs with high ISE scores for the desired AGANT and low scores (usually < 0) for “anti-targets” (targets/actions that are important to avoid).

#### 2.5.1. Cannabinoid 2 Receptor (CB2R)

The CB2R agonists hold promise as a new class of therapeutics for indications as diverse as pain, neuroinflammation, Alzheimer’s disease (AD), immune suppression, osteoporosis, cancer, several CNS disorders, including drug addiction and anxiety, liver disease, and more [33]. These potential indications are supported by strong preliminary data from multiple investigators using diverse preclinical models. CB2 agonists modulate central neuroinflammatory conditions, modify opioid-induced tolerance and reward-seeking behavior, and modulate peripheral neuroinflammation [34], avoiding the adverse psychotropic effects accompanying CB1R.

Eighty-two drugs from different indications were predicted to be agonists at the CB2R (score ≥ 0.7). If we also require those agonists to be devoid of anti-targets, CB1R, and CB2R antagonism (scores < 0), we have 61 drugs, 32 of which are approved drugs (see Table S3). The set of 82 drugs is diverse and has a low similarity to the known agonists (average Tanimoto = 0.13, Table 3). All except one drug (Alectinib, ISE score = 0.82) docked to the CB2R structure, with docking scores ranging from −4.3 to −12 Kcal/mol (Table S3). The majority (60 drugs) have a docking score of ≤−8 Kcal/mol, compared to the agonist LEI-102 (from the PDB structure- 8GUT [35]), which has a docking score of −8.2 Kcal/mol.

**Figure 2:**
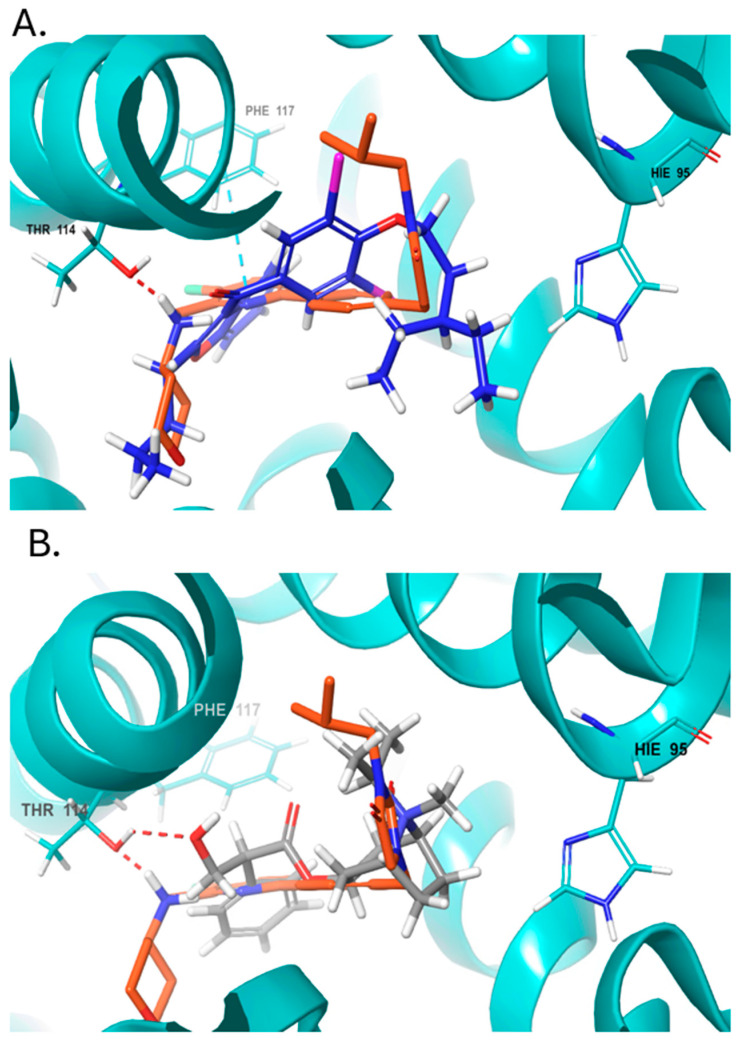
Docking positions at the CB2R agonist pocket. (**A**) Amiodarone (blue docking score = −12 Kcal/mol) aligned with native agonist LEI-102 (orange). (**B**) Ipratropium bromide (gray, docking score = −10.5 Kcal/mol) aligned with native LEI-102 (orange). H-bonds are shown as dashed red lines, pi-pi stacking as teal dashed lines.

Amiodarone (DB01118) is an antiarrhythmic drug with the highest docking score of −12 Kcal/mol (ISE score = 0.74, Figure 2A). Ipratropium bromide (DB00332, ISE score = 0.83), an anticholinergic drug used in the control of symptoms related to bronchospasm in chronic obstructive pulmonary disease (COPD), also has a higher docking score than the native ligand with −10.5 Kcal/mol (Table S3). Both are found by docking to interact with key residues at the CB2R pocket, such as F87^2.57^, F91^2.61^, F94^2.64^, H95^2.65,^ F117^3.36^, and V261^6.51^ [35,36]. Ipratropium bromide also makes an H-bond with T114^3.33^ (Figure 2B). Interestingly, some local anesthetics, such as Ropivacaine, Dyclonine, and Bupivacaine, got high scores as CB2 agonists, but their scores were not in the range of <−8 Kcal/mol in docking.

#### 2.5.2. Histamine 3 Receptor (H3R) 

Due to its widespread distribution and its ability to affect multiple neurotransmitter systems (including histamine, dopamine, serotonin, acetylcholine, and norepinephrine), the modulation of H3R activity has been proposed for a broad range of indications such as AD, attention deficit hyperactivity disorder (ADHD), sleep disorders, pain, and obesity [37]. Several preclinical studies showed that H3R antagonists reduced food intake, body weight, and blood glucose levels in obese animals [38]. Two H3R antagonists, SCH-497079 (NCT00642993, NCT00673465) and HPP-404 (NCT01540864), were evaluated in clinical trials for treating obesity and diabetes. However, these molecules were not developed further due to low efficacy [39]. Looking for potential H3R antagonists (with no agonism) among the screened drugs, we found 109 candidates (Table S4). Approximately 40% of them docked with a high docking score (less than −8 Kcal/mol).

**Figure 3:**
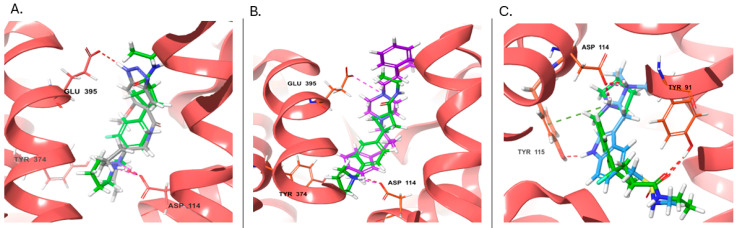
Docking poses at the H3R (red). The native antagonist, PF-03654746, is presented as green sticks aligned to the different docked drugs: (**A**) Alizapride (DB01425, gray sticks); (**B**) Eprazinone (DB08990, violet sticks); (**C**) Sumatriptan (DB00669, azure sticks). Hydrogen bonds, salt bridges, and pi-cation interactions are shown as red, pink, and green dashed lines, respectively.

Alizapride (DB01425), a D2 antagonist used in treating nausea and vomiting, including that which may occur postoperatively, has a high ISE score of 0.9 and docking score of −8.8 Kcal/mol. The docked alizapride has hydrogen bonds with D114^3.32^ (also a salt bridge) and E395^7.36^ and interacts with the hydrophobic pocket constituted by Y115^3.33^, Y374^6.51^, F398^7.39^, and W402^7.43^ (Table S4, Figure 3A). Eprazinone (DB08990), a mucolytic agent, has a higher docking score (−11.6 Kcal/mol) with similar interactions as alizapride and PF-03654746 (native ligand, Figure 3B). Another example is Sumatriptan (DB00669, a serotonin receptor agonist), used to treat migraines and cluster headaches (ISE score = 0.9, docking score = −11.6 Kcal/mol). Sumatriptan docked to the H3R structure and forms hydrogen bonds with Y91^2.61^, D114^3.32^, and Y115^3.33^ (Figure 3C).

#### 2.5.3. Histamine 1 and 4 Receptors (H1R, H4R)

The H4R mediates lung function and inflammation in animal asthma models and mediates pruritic responses [40]. Antihistamines that target the H1R are effective in reducing acute pruritus but are ineffective in pruritus experienced by patients with atopic dermatitis. Antagonists of the H4R reduce pruritus in several conditions. The anti-pruritic effect of H4R antagonists has recently been shown in human clinical studies, validating the preclinical findings in animal models. A selective H4R antagonist inhibits histamine-induced pruritus in health volunteers and reduces pruritus in patients with atopic dermatitis [40]. The antagonism of histamine H1 and H4 receptors ameliorates chronic allergic dermatitis via anti-pruritic and anti-inflammatory effects in mice models [41]. It may provide superior relief of signs and symptoms of allergic conjunctivitis compared to traditional allergy therapies [42].

**Figure 4:**
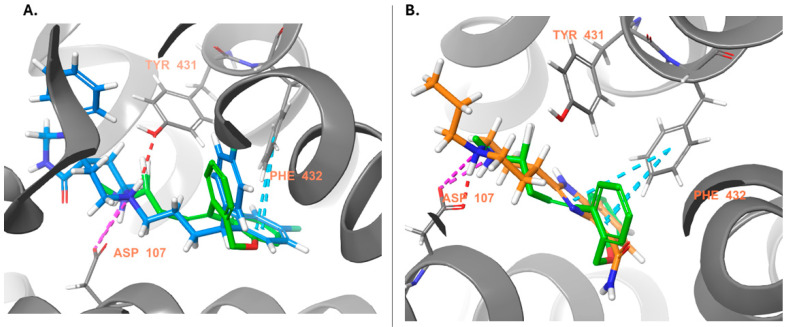
Docking poses at the H1R (gray). The native antagonist, Doxepin, is presented as green sticks aligned to the docked drugs: (**A**) Fluspirilene (DB04842, azure sticks); (**B**) DB07330 (orange sticks). Hydrogen bonds, salt bridges, and pi-pi stacking are shown as red, violet, and azure dashed lines, respectively.

Looking for H1/H4 antagonists yields only two drugs (Table S5). Both drugs are diverse compared to the known antagonists of H1 and H4 receptors (Table 3). Fluspirilene (DB04842, H1 antagonist score = 0.92, H4 antagonist score = 0.84) is an approved antipsychotic agent used in the treatment of schizophrenia through D2/5-HT2A antagonism. Fluspirilene overlaps with the doxepin pose in the H1R binding pocket and has a salt bridge with D107^3.32^ and a hydrogen bond with Y431^6.51^ (Figure 4A). The fluorobenzene rings form pi-pi stacking with Y108^3.33^, W428^6.48^, F432^6.52^, and F435^6.55^. In addition, it interacts with the anion-binding residues K179^ECL2^ and K191^5.39^ (Figure 4A). However, it got a very low docking score (−2.1 Kcal/mol, Table S5), which could be because of the extended binding site towards the extracellular loop 2 (ECL2). DB07330, an experimental drug, was explored for its potential as a poly (ADP-ribose) polymerase inhibitor for cancer treatment [43]. DB07330 belongs to the benzimidazole compounds and has a high docking score of −10.5 Kcal/mol. The docked pose retrieved the key interactions with the H1R (Figure 4B).

#### 2.5.4. Dopamine 3 Receptor (D3R)

D3Rs are a primary focus in the design and development of therapeutics for dopamine-related disorders. Based on sequence homology, they belong to the D2-like receptors (D2, D3, and D4). D3R is less abundant than the D2 subtype and has different tissue localization, such as nucleus accumbens, thalamus, hippocampus, and cortex, which is considered important for psychotic symptoms [44]. There is strong evidence that D3 receptor antagonists could be effective antipsychotic agents and could also be involved in behavioral sensitization, with potential efficacy in the treatment of drug abuse—tobacco, opioid, and psychostimulant use disorders [44,45].

**Figure 5:**
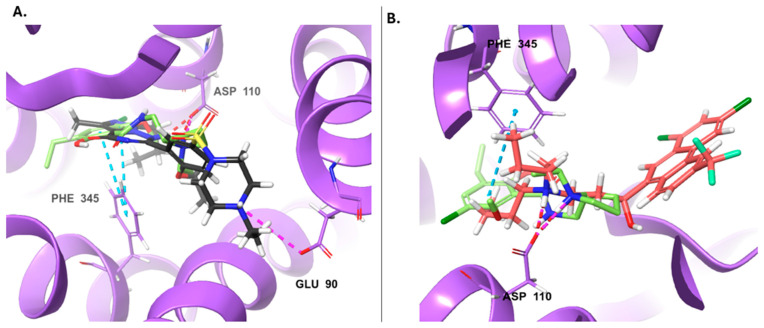
Docking poses at the D3R (violet). The native antagonist, Eticlopride, is presented as lime sticks aligned to the docked drugs: (**A**) Vardenafil (DB00862, dark gray sticks); (**B**) Halofantrine (DB01218, red sticks). Hydrogen bonds, salt bridges, and pi-pi stacking are shown as red, violet, and azure dashed lines, respectively.

The peculiar distribution and low brain abundance of D3 receptors make them a valuable target for developing drugs devoid of motor side effects classically caused by D2 antagonists. However, the close homology with the D2 receptor subtype makes developing D3-selective antagonists challenging. We found 36 molecules that have the potential to act as D3 antagonists (Table S6). If we also consider the D2 antagonist as anti-target (requiring a score < 0), only 12 molecules are left, but some may have D2 agonism, although few have low scores, like Sildenafil and Halofantrine. So, if we exclude these molecules, only one drug is left: Vardenafil (DB00862), a phosphodiesterase 5 inhibitor used to treat erectile dysfunction. However, vardenafil got a low docking score (−4.9 Kcal/mol) to D3R, despite it formed an H-bond with D110^3.32^ and E90^2.64^ and hydrophobic contacts like the native antagonist (eticlopride, see results Section 2.4. for the detailed residues [32]) (Figure 5A). Halofantrine (DB01218) is an antimalarial drug that has a high D3 antagonist score, a low score at the D2 agonist model, but a better docking score than vardenafil (0.89, 0.15, and −6.8, respectively, Table S6). It was docked into the D3R pocket and formed a salt bridge with D110^3.32^ and hydrophobic interactions with V111^3.33^, I183^ECL2^, F345^6.51,^ and F346^6.52^ (Figure 5B). Both predicted drugs are larger than the native ligand.

## 3. Materials and Methods

### 3.1. Iterative Stochastic Elimination (ISE) Models for Selected GPCRs

Models were constructed using the “Iterative Stochastic Elimination” (ISE) algorithm [21,22], which is primarily used to build classification models of molecular bioactivity. These models are used for screening databases to identify novel and diverse bioactive candidates [46,47].

ISE is a ligand-based method; the learning set comprises reported molecules from the ChEMBL database, agonists with EC_50_ values, or antagonists with IC_50_ or K_i_ values at the different human GPCRs (values less than 100 µM) [27]. Duplicates (with the same ChEMBL ID) and drugs from the DrugBank database were excluded.

The decoys were selected from the ZINC druglike database [48]. The decoys (with a ratio of 1:100 or 1:1000 to the known molecules from ChEMBL) are selected based on the applicability domain of the known molecules [49]. The learning set was prepared, and 2D descriptors were calculated using MOE software (v. 2011.10) [50]. Based on the calculated descriptors, we removed mutagenic and reactive molecules from the learning sets (indicated by the presence or absence of potentially toxic and reactive groups, respectively).

The ISE algorithm generates a large number of filters, each representing a combination of molecular property ranges, which best distinguish between two classes: agonist vs. non-agonist or antagonist vs. non-antagonist.

The MCC [23] is a metric used to evaluate the quality of binary classifications. It is particularly advantageous for datasets with a significant class imbalance, as it is not biased towards the majority class. The MCC considers all four components of the confusion matrix: True Positives (TP)—correctly predicted positive instances, True Negatives (TN)—correctly predicted negative instances, False Positives (FP)—incorrectly predicted positive instances, False Negatives (FN)—incorrectly predicted negative instances. MCC values range from −1 to 1, producing high scores only if the model performs well in all four categories of the confusion matrix.

$$MCC=\frac{TP\times TN-FP\times FN}{\surd (TP+FP)(TP+FN)(TN+FP)(TN+FN)}$$

The AUC is another performance measurement for classification problems at different classification thresholds. The ROC curve plots the true positive rate (TPR) against the false positive rate (FPR).

$$TPR\left(Sensitivity\right)=\frac{True\;Positives}{Total\;Positives}=\frac{TP}{TP+FN}$$

$$TNR\left(Specificity\right)=\frac{True\;Negatives}{Total\;Negatives}=\frac{TN}{TN+FP}$$

Screening any set of molecules through ISE models allows ranking them by their ability to pass through the model. A molecule is assigned a positive weight if it satisfies all filter criteria and a negative weight otherwise. The final score is calculated as the average of these weights across all filters. The F-score, a metric combining precision and recall, evaluates the model’s accuracy in predicting active compounds.

$$Score=\frac{\sum_{i=1}^{n}\delta_{positive}F_{positive(i)}-\delta_{negative}F_{negative(i)}}{n}$$

The final scores are between −1 and +1. A higher score indicates a greater likelihood of experimental successful discovery for a molecule. We determine a suitable index threshold based on enrichment factor and true positive/false positive rates to filter large compound libraries efficiently.

### 3.2. Drugs Database

We downloaded the DrugBank database )DB) (July 2016, version 4.5) [20], which contains 7130 drug entries. Using MOE software (v.2011.10) [50], we apply “Molecular Database Wash” [51] and 2D descriptors calculation for virtual screening (VS) of the DrugBank DB through our models.

### 3.3. Drug-Protein Matrices

The total reported actions (agonist and antagonist) between the drugs and the 31 GPCRs used in this study are 1212 of 361 drugs [19]. The data was collected from: (1) DrugBank version 5.1.9, access date: Jan/2022; (2) ChEMBL27 (access date: November 2020) [27]; (3) Therapeutic Target Database (TTD, access date: November 2020) [28].

Drug-target adjacency matrices were created for the reported and predicted AGANT by ISE models. Denoting the target receptor set as T = {t1, t2, …, tm} and the drug set as D = {d1, d2, …, dn}, the drug-target (DT) binary interactions can be described as a bipartite DT graph G(D, T, E), where E = {eij: di ∈ D, tj ∈ T}. A link is drawn between di and tj only if the drug di has any action with the target tj. The DT bipartite network can be presented by an n × m adjacency matrix {aij}, where aij = 1 when di and tj interact; otherwise, aij = 0. Based on the reported AGANT, there are 1165 reported Drug-GPCR interactions/connections between 361 drugs (out of the 7130 DrugBank DB) and 31 GPCRs [19].

### 3.4. Tanimoto Fingerprint Similarity

The ECFP4 fingerprint was used to compute fingerprint similarity between the different molecule sets by the rdkit.DataStructs.FingerprintSimilarity module (RDKit toolkit v. ‘2024.03.5’ [52]), with Python 3.9.13.

### 3.5. Docking

#### 3.5.1. Structures Selection

The selected structures for each receptor were downloaded from the PDB [53] and are listed in Table 6. The overall structures of the four complexes of CB2R that were published recently [35], were comparable (CB_2_R-LEI-102-G_i_-scFv16, CB_2_R-APD371-G_i_-scFv16, CB_2_R-HU308-G_i_-scFv16, and CB_2_R-CP55,940-G_i_-scFv16, complex, at 2.9, 3.0, 3.0, and 2.9 Å, respectively), with RMSD of the Cα atoms of the receptors around 0.35 Å [35]. The ligand binding interfaces of the four CB_2_R and G_i_ complexes were similar to each other and to those of the previous AM12033-CB_2_R-G_i_ or WIN55212-2-CB_2_R-G_i_ complex structures [35]. We chose the complex 8GUT with the LEI-102—agonist.

For H3R and H1R, only one structure is available with antagonist binding, 7F61 [30] and 3RZE [31], respectively. H4R has no resolved structure yet. The H1R structure contains a mixture of E and Z isomers, and we continue with the E isomer in the docking studies. For D3R, the only structure with an antagonist is 3PBL [32]. We chose chain A to perform the docking.

#### 3.5.2. Ligand and Protein Preparation

Molecule sets and redocked ligands were prepared for the docking using Schrödinger’s LigPrep tool (Schrödinger release 2021–3 [54]). The process includes energy-minimization using the OPLS4 force field, generating possible states at pH 7.0 ± 0.2 by Epik, generating tautomers and desalt.

The four GPCR structures were optimized and prepared using the ‘Protein Preparation’ wizard in Maestro Schrödinger [55] with the following options: cap termini, fill missing side chains, generate het states in pH 7.4 ± 2, the metal ionization state was corrected, and we deleted all water molecules if there were any. Hydrogens were added and minimized with an OPLS4 force field. In the H1R structure, we deleted the phosphate ions as well, even though it affects the binding of some ligands and the stability of H1R [31]. We also kept isomer E for the grid generation and docking. We kept chain A for the D3R (3PBL [32]) to perform the docking.

#### 3.5.3. Grid Generation and Molecular Docking

The grid box was defined around the ligand binding site in each selected structure, using Glide’s ‘Receptor Grid Generation’ tool (Schrödinger release 2021–3 [56]). Glide’s extra precession (XP) docking with flexible ligand sampling was performed.

## 4. Conclusions

GPCRs are the family of proteins most frequently targeted by approved drugs. Applying an existing drug to a new indication promises rapid clinical impact at a lower cost than de novo drug development. Of the approved GPCR-targeted drugs, 33% have more than one indication, and the overall average is 1.5 indications per drug [57]. Drug repurposing presents a promising strategy for optimizing the therapeutic potential of existing medicines, quickly identifying effective treatments, and effectively addressing challenging diseases. Computational methods play a crucial role in accelerating the drug repurposing process and potentially revolutionizing the field of drug discovery.

We looked for unknown/new AGANT to suggest repurposing opportunities involving the six selected GPCR families. The intricate network of drug-target interactions underscores the complexity and potential of polypharmacology in drug discovery and repurposing. Our models cover a specific group of receptors and are not a systemic screening platform.

The screening results identified numerous new actions, which we confirmed by docking for CB2, H1/3/4, and D3 receptors, potentially addressing unmet medical needs in neuroinflammation, obesity, allergic dermatitis, and CNS disorders. It is important to remember that docking has no implication on the action at a receptor but only on the chance of binding, which can result in agonism or antagonism, aka AGANT. However, high-scored molecules will not always be optimal for repurposing as some factors need to be considered, like the route of administration, the primary indication, and the safety profile. For example, tetracaine (DB09085) is predicted to be an H3R antagonist. However, it will not be a choice as it is used as a local anesthetic in the eyes and skin during medical procedures. Amiodarone (DB01118), an antiarrhythmic drug, will not be used for neuroinflammation. Moreover, a new indication may have underlying mechanisms that the repurposed drug does not address effectively. Finding the optimal dosage and administration route for the new indication may be challenging, potentially leading to insufficient drug exposure at the target site.

Future work should focus on experimental validation of these computational predictions and further exploration of repurposing opportunities to expedite the development of effective treatments for various diseases. Integrating computational methods into the drug discovery pipeline holds promise for enhancing drug development efficiency and expanding therapeutic options.

## Figures and Tables

**Table 1:** The number of successful activities predicted by the ISE models and the new predicted activities in different cutoff scores obtained by DrugBank molecules.

Cutoff Score	Number of Successful Activity Predictions	Number of New Activities	% of Successful Predictions
>0	684	21,987	56%
≥0.1	648	19,052	53%
≥0.2	605	16,230	50%
≥0.3	555	13,882	46%
≥0.4	516	11,687	43%
≥0.5	466	9660	38%
≥0.6	419	7661	35%
≥0.7	375	5982	31%
≥0.8	307	4407	25%
≥0.9	164	1517	14%

**Table 2:** The number of successful interactions/connections predicted by the ISE models and the new predicted interactions in different cutoff scores obtained by DrugBank molecules.

Cutoff Score	Number of Successful Interaction Predictions	Number of New Interactions	% of Successful Predictions
>0	805	17,595	69%
≥0.1	771	15,414	66%
≥0.2	724	13,246	62%
≥0.3	671	11,391	58%
≥0.4	626	9695	54%
≥0.5	571	8137	49%
≥0.6	515	6486	44%
≥0.7	459	5120	39%
≥0.8	381	3870	33%
≥0.9	202	1330	17%

**Table 3:** Average Tanimoto similarity values for the different receptors. The Tanimoto values were measured in three sets for each receptor: Predicted drugs vs. known AGANT (P-A), predicted drugs vs. predicted drugs (P-P), and known AGANT vs. known AGANT (A-A).

Receptor	Number of Known Actives Used to Build the Model	Number of Predicted Drugs	Average Tanimoto P-A	Average Tanimoto P-P	Average Tanimoto A-A
CB2R	275	82	0.13	0.15	0.18
H3R	474	109	0.13	0.16	0.20
H1R	95	2	0.14	0.17	0.21
H4R	169	2	0.14	0.17	0.19
D3R	269	36	0.13	0.14	0.23

P—Predicted drugs; A—AGANT compounds. CB2R—Cannabinoid 2 receptor; H3R—Histamine 3 receptor; H1R—Histamine 1 receptor; H4R—Histamine 4 receptor; D3R—Dopamine 3 receptor

**Table 4:** Redocking results of the native ligands in each structure. The presented results are the docking score, the buried surface area (BSA, Å^3^), and the number of hydrogen bonds and contacts for the best-docked pose. The last column presents the RMSD values for the docked vs. the crystal structure pose.

Receptor	Ligand	Docking Score (Kcal/mol)	BSA (Å^3^)	Number of H-Bonds	Number of Contacts	RMSD (Å)
CB2R	LEI-102 CB2R agonist	−8.2	1061	1	514	2.6
H3R	PF-03654746 H3R antagonist	−12.8	844	3	600	2.8
H1R	Doxepin (E isomer) (H1R antagonist)	−12	731	1	379	2.5
D3R	Eticlopride (D2R/D3R antagonist)	−7.9	815	1	319	3.8

CB2R—Cannabinoid 2 receptor; H3R—Histamine 3 receptor; H1R—Histamine 1 receptor; D3R—Dopamine 3 receptor; H-Bonds—Hydrogen bonds; RMSD—Root Mean Square Deviation.

**Table 5:** Docking results of the predicted drugs in each structure. The presented results are the number of predicted drugs in each set, the number of successfully docked ones and their docking score, the buried surface area (BSA), and the number of hydrogen bonds and contacts. The results presented are for the best-docked pose in each set.

Receptor	Number of Predicted Drugs	Number of Docked Drugs	Docking Score (Kcal/mol)	BSA (Å^3^)	Number of H-Bonds	Number of Contacts
CB2R	82	81	−4.3–−12	652–1054	0–2	215–457
H3R	109	90	9.1–−11.9	627–1099	1–5	516–869
H1R	2	2	−2.1–−10.5	780–1082	1	307–559
D3R	36	36	−2.2–−9.7	799–1381	0–4	284–480

CB2R—Cannabinoid receptor; H3R—Histamine 3 receptor; H1R—Histamine 1 receptor; D3R—Dopamine 3 receptor; H-Bonds—Hydrogen bonds.

**Table 6:** The selected GPCR structures were used for docking studies.

Receptor	Structure	Ligand	Reference
Cannabinoid receptor 2 (CB2R)-Gi-Scfv16 complex	8GUT Cryo-EM2.98 Å	LEI-102 (CB2R agonist) pEC_50_ = 6.9 ± 0.2 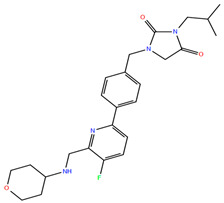	[35]
Histamine 3 receptor (H3R)	7F61 X-ray 2.6 Å	PF-03654746 (H3R antagonist) IC_50_ = 1.45 ± 0.51 (nM) 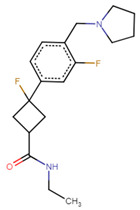	[30]
Histamine 1 receptor (H1R)-T4-lysozyme fusion protein complex	3RZE X-ray 3.1 Å	Doxepin (E isomer) (H1R antagonist) K_i_ = 2.2 ± 0.5 (nM) 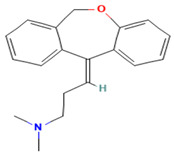	[31]
Dopamine 3 receptor (D3R)-T4-lysozyme fusion protein complex	3PBL X-ray 2.89 Å	Eticlopride (D2R/D3R antagonist) K_i_ = 0.16 (nM) K_i_ (D2R) = 0.5 (nM) 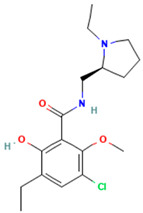	[32]

## Data Availability

Any further data can be requested from the authors. Email: shayma.el-atawneh@mail.huji.ac.il.

## Supplementary Materials

The following supporting information can be downloaded at: https://www.mdpi.com/article/10.3390/ijms251810230/s1.
