# Supplementary material for: A Machine Learning Algorithm Suggests Repurposing Opportunities for Targeting Selected GPCRs
Source: Int J Mol Sci. 2024 Sep 23;25(18):10230. doi: 10.3390/ijms251810230 (PMC11432050; doi:10.3390/ijms251810230)
Supplement: Supplementary file 1 [file ijms-25-10230-s001.zip › Supplmentary_figures.pdf]

Supplementary figures

A Machine Learning Algorithm Suggests Repurposing Opportunities for Targeting Selected GPCRs

Shayma El-Atawneh and Amiram Goldblum *

Molecular Modelling and Drug Design Lab, Institute for Drug Research and Fraunhofer Project Center for Drug Discovery and Delivery, Faculty of Medicine, The Hebrew University of Jerusalem,

Jerusalem 9112001, Israel; shayma.el-atawneh@mail.huji.ac.il

***Correspondence: amiramg@ekmd.huji.ac.il**

Figure S1. Chemical structures of predicted drugs.

A.

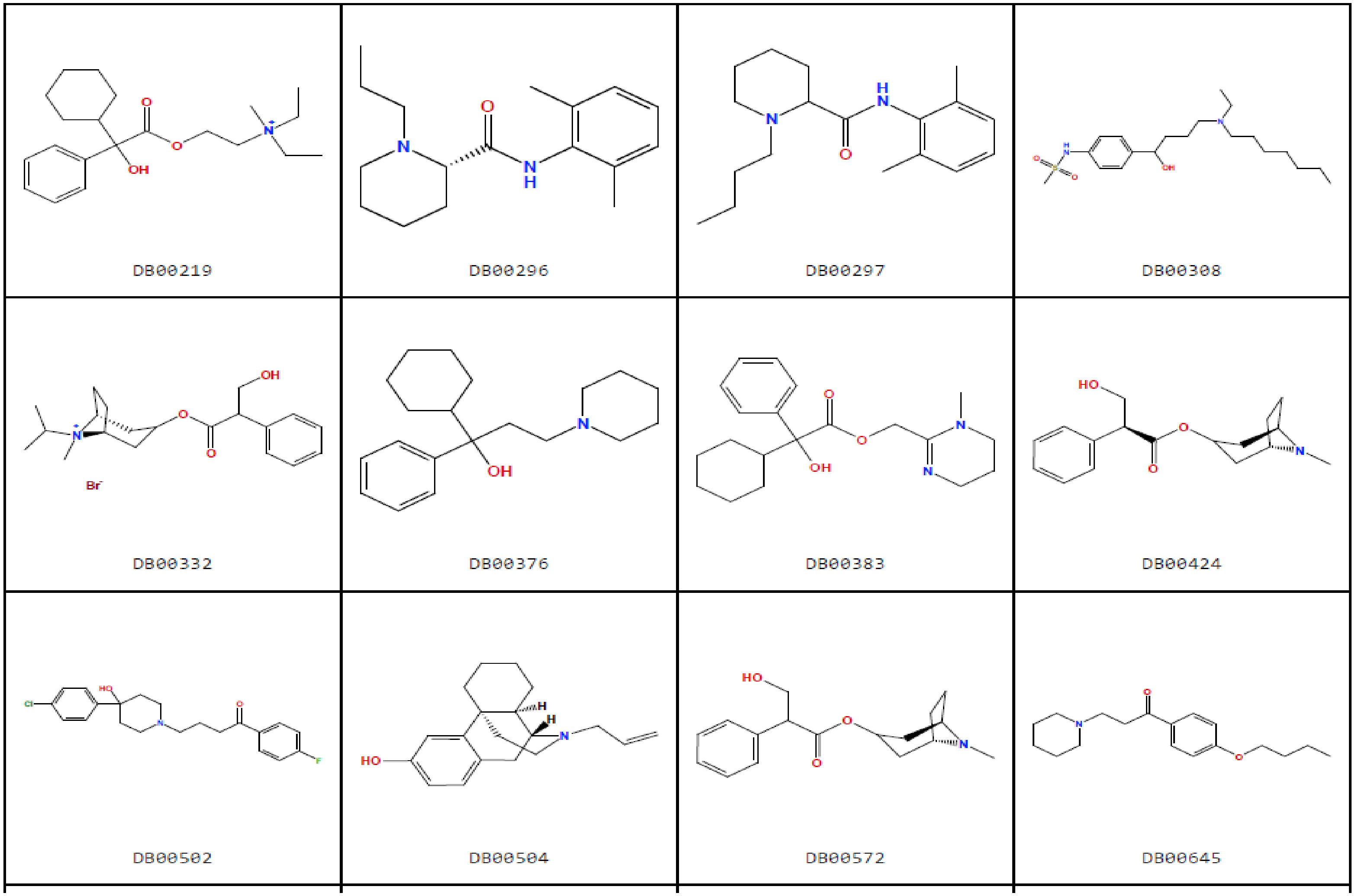

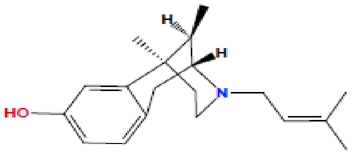 DB00652	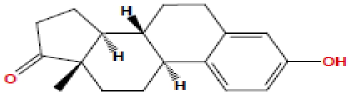 DB00655	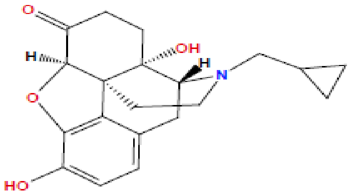 DB00704	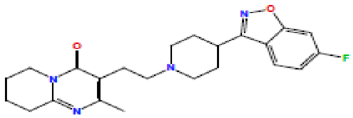 DB00734
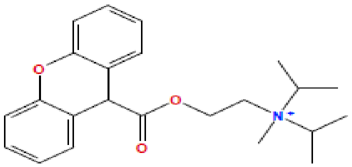 DB00782	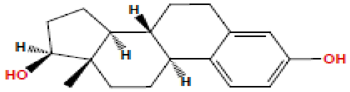 DB00783	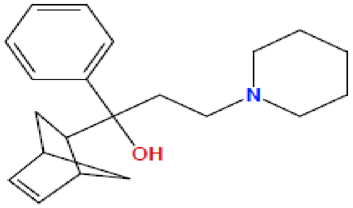 DB00810	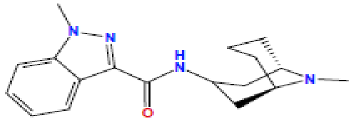 DB00889
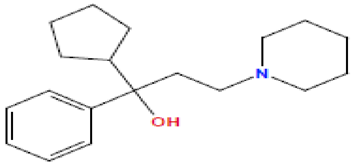 DB00942	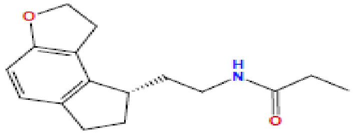 DB00980	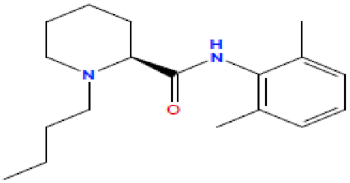 DB01002	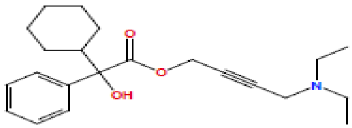 DB01062

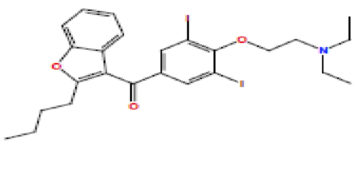<br>DB01118	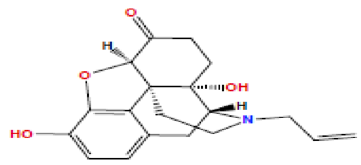<br>DB01183	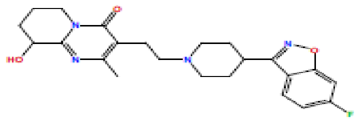<br>DB01267	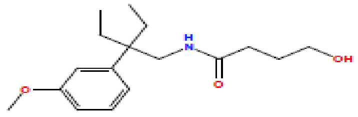<br>DB01487
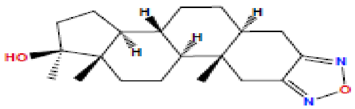<br>DB01514	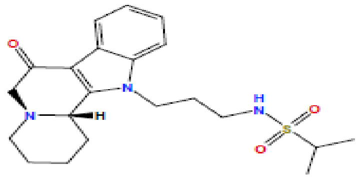<br>DB01967	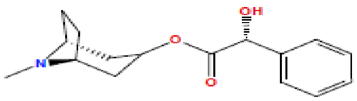<br>DB02161	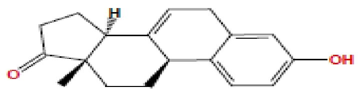<br>DB02187
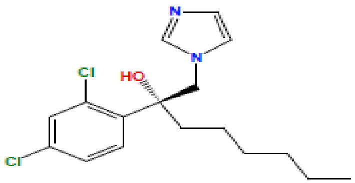<br>DB02617	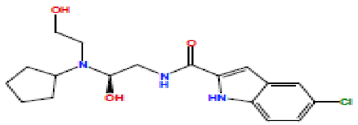<br>DB03288	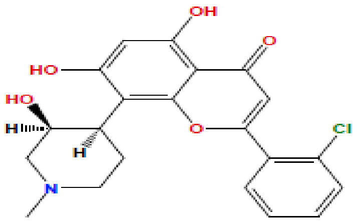<br>DB03496	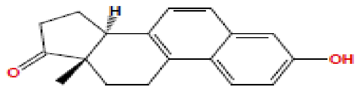<br>DB03515

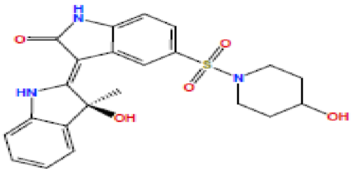<br>DB03583	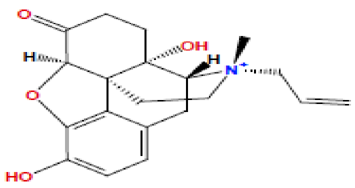<br>DB04509	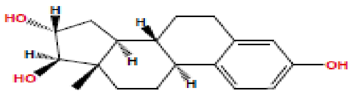<br>DB04573	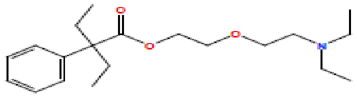<br>DB04822

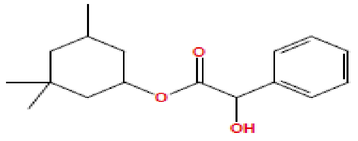 DB04838	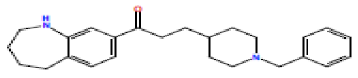 DB04859	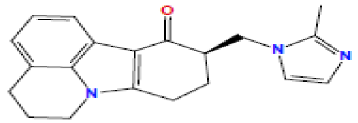 DB04885	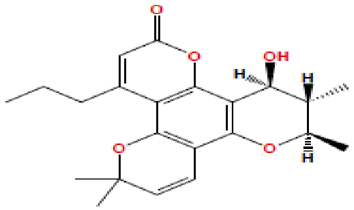 DB04886
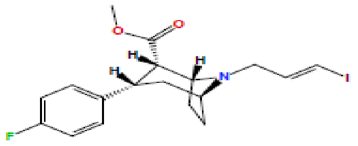 DB04947	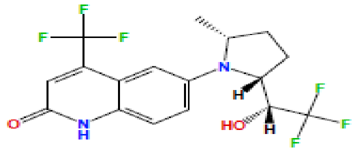 DB05234	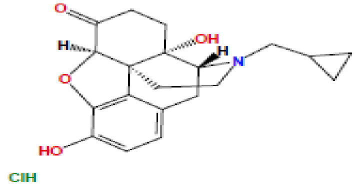 DB05302	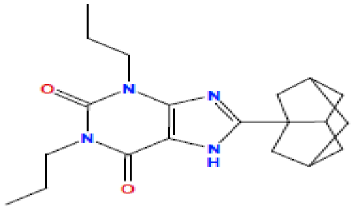 DB05360
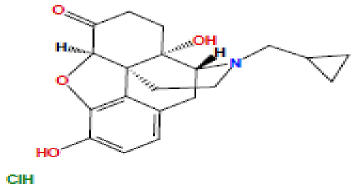 DB05508	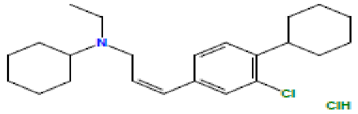 DB05792	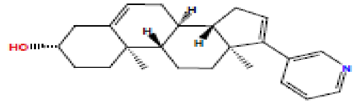 DB05812	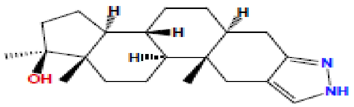 DB06718
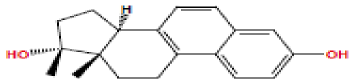 DB06871	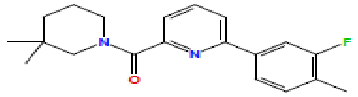 DB06992	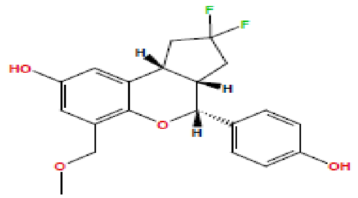 DB07036	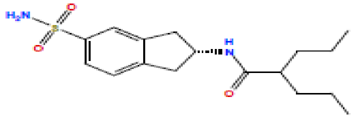 DB07048

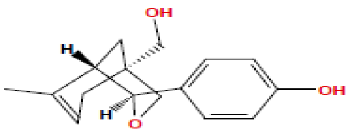<br>DB07086	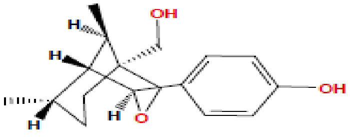<br>DB07087	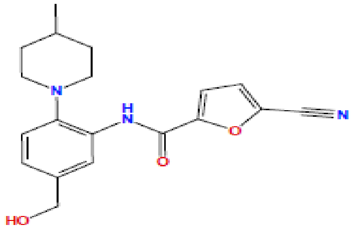<br>DB07167	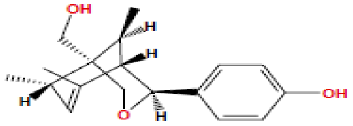<br>DB07195

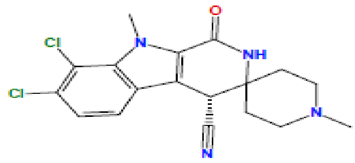<br>DB07242	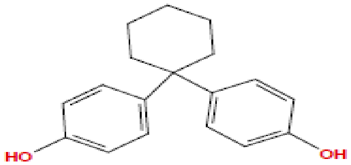<br>DB07485	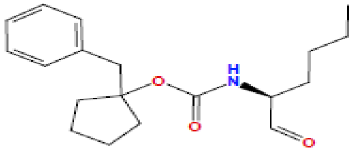<br>DB07593	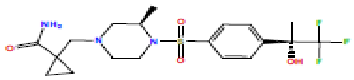<br>DB07624
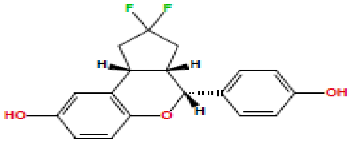<br>DB07638	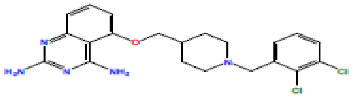<br>DB07643	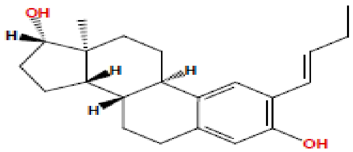<br>DB07678	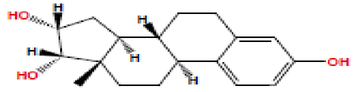<br>DB07702
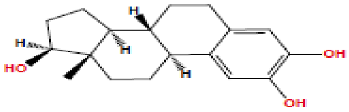<br>DB07706	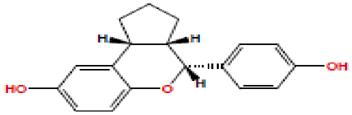<br>DB07933	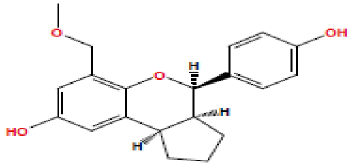<br>DB08020	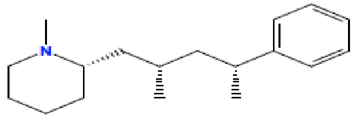<br>DB08071

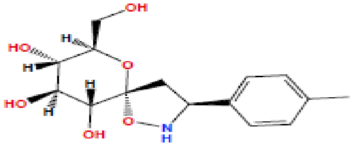 DB08503	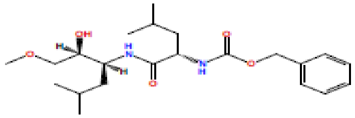 DB08526	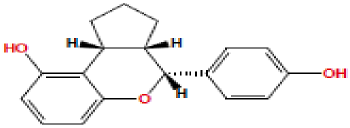 DB08737	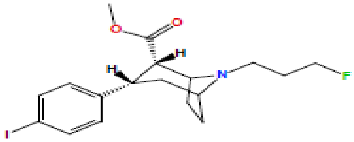 DB08824
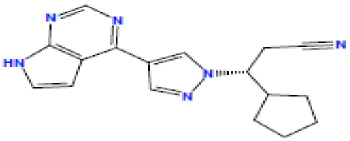 DB08877	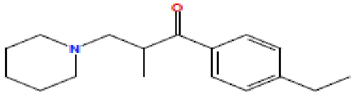 DB08992	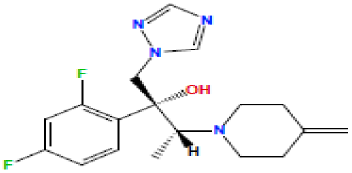 DB09040	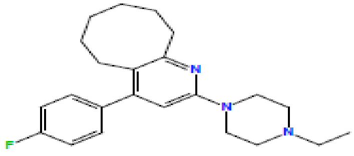 DB09223

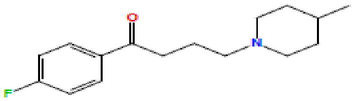 DB09224	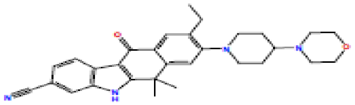 DB11363

B.

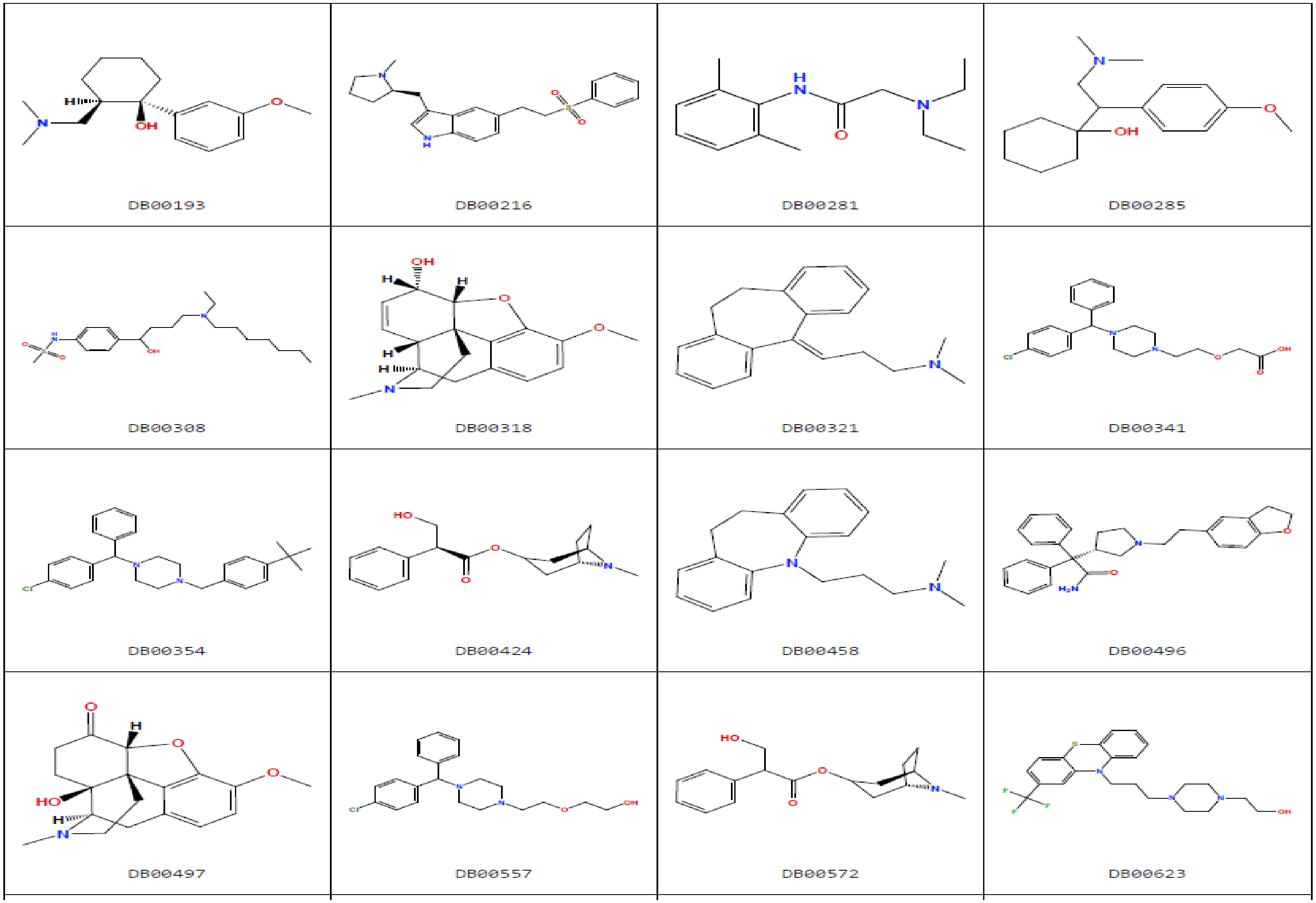
DB00193
DB00216
DB00281
DB00285
DB00308
DB00318
DB00321
DB00341
DB00354
DB00424
DB00458
DB00496
DB00497
DB00557
DB00572
DB00623

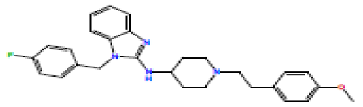

DB00637

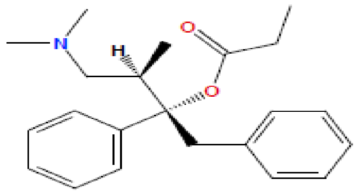

DB00647

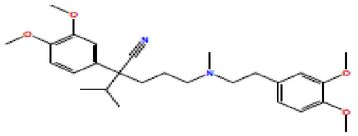

DB00661

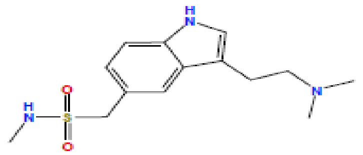

DB00669

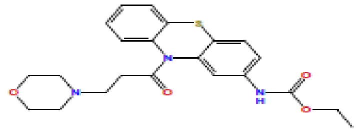

DB00680

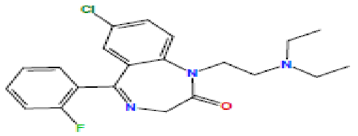

DB00690

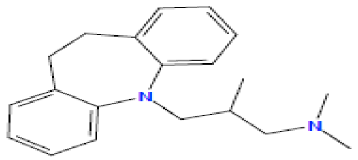

DB00726

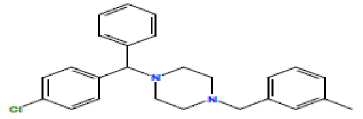

DB00737

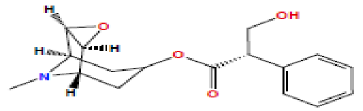

DB00747

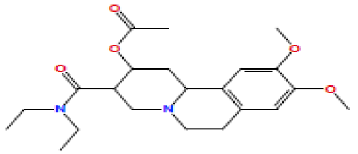

DB00767

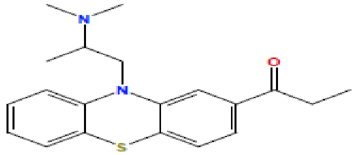

DB00777

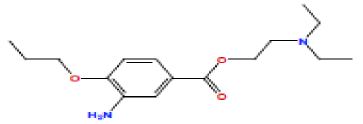

DB00807

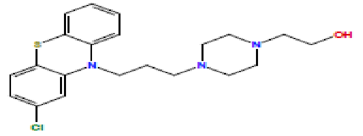

DB00850

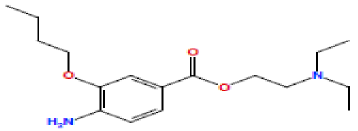

DB00892

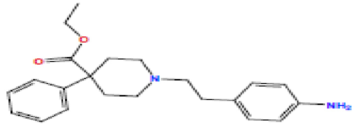

DB00913

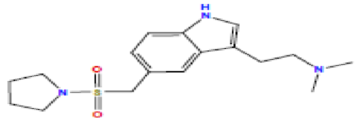

DB00918

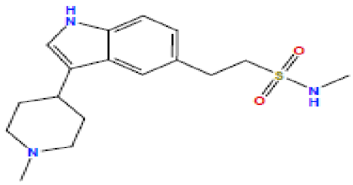 DB00952	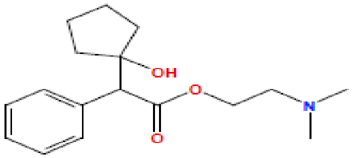 DB00979	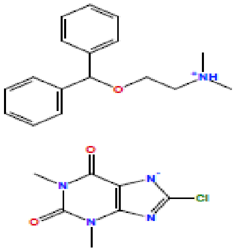 DB00985	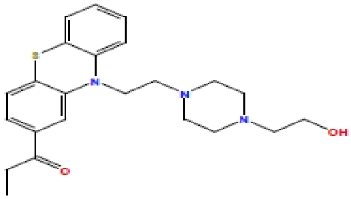 DB01038
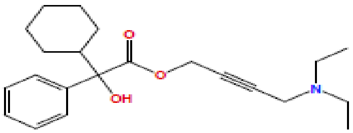 DB01062	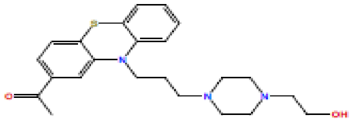 DB01063	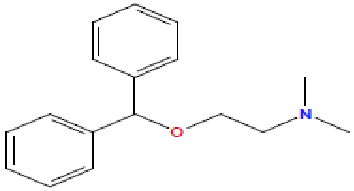 DB01075	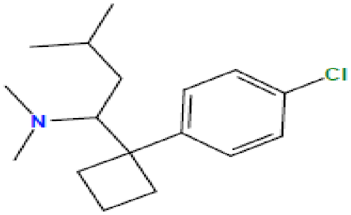 DB01105

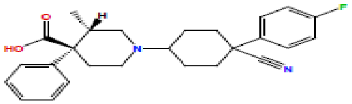 DB01106	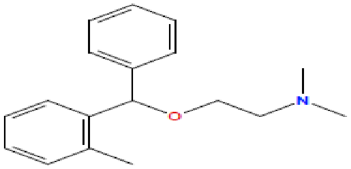 DB01173	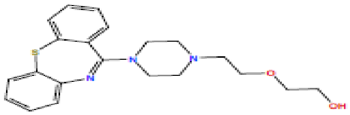 DB01224	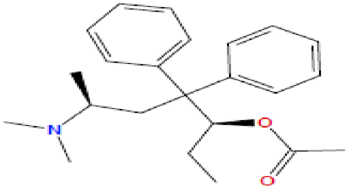 DB01227
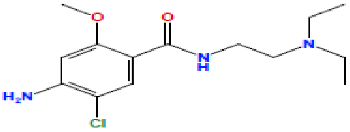 DB01233	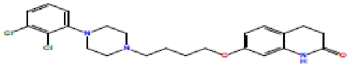 DB01238	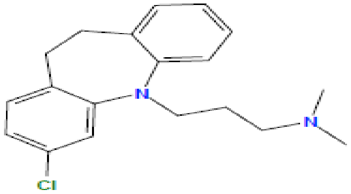 DB01242	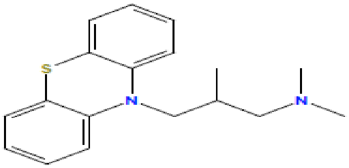 DB01246

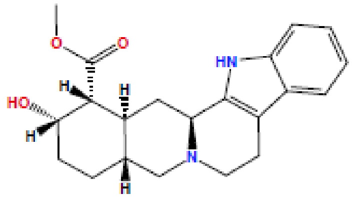 DB01392	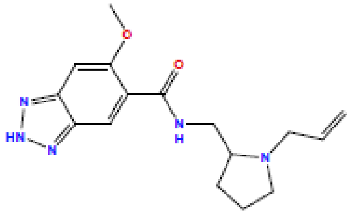 DB01425	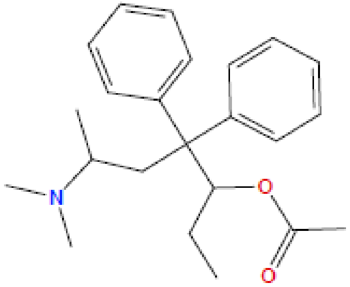 DB01433	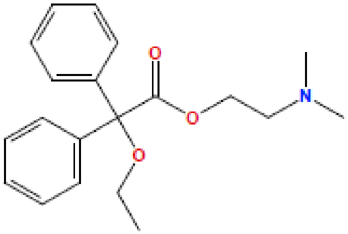 DB01461
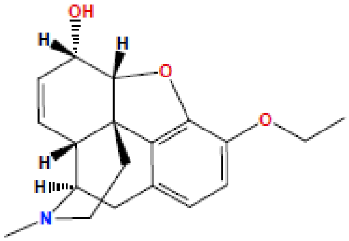 DB01466	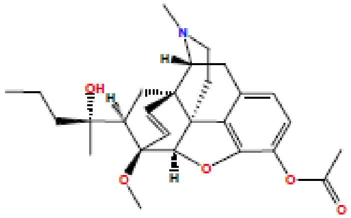 DB01469	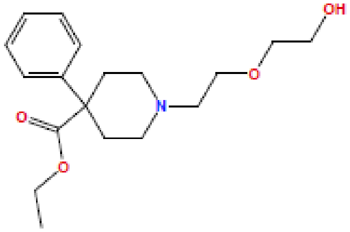 DB01505	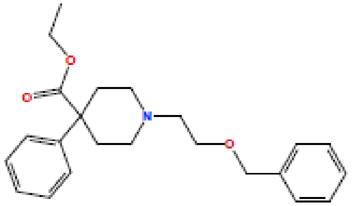 DB01518
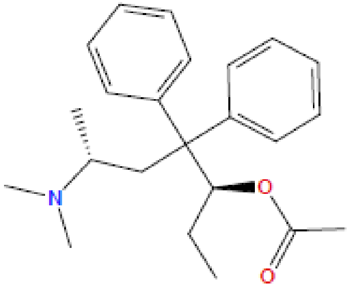 DB01522	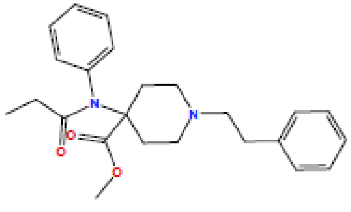 DB01535	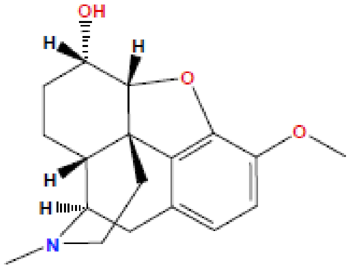 DB01551	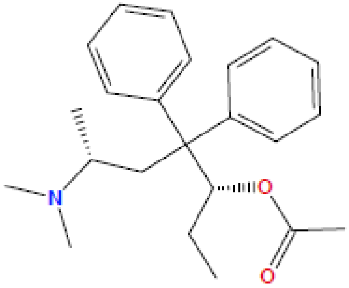 DB01555

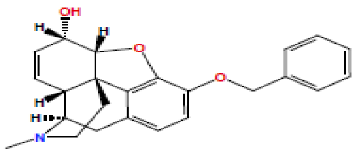<br>DB01573	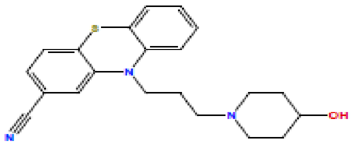<br>DB01608	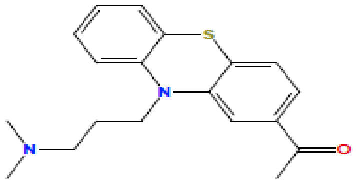<br>DB01614	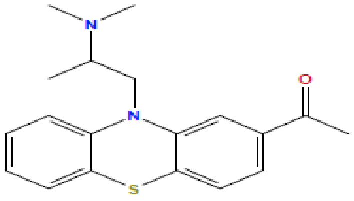<br>DB01615
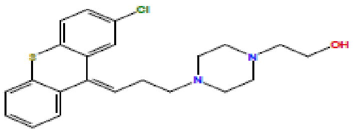<br>DB01624	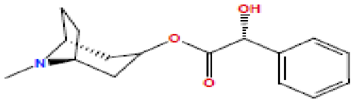<br>DB02161	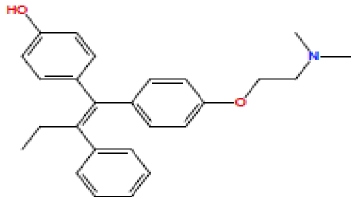<br>DB04468	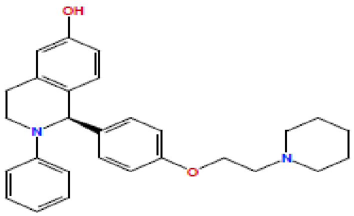<br>DB04471
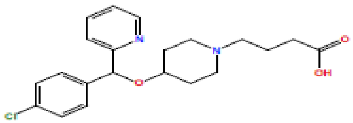<br>DB04890	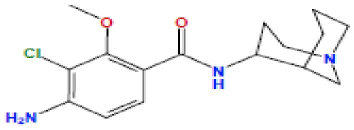<br>DB04917	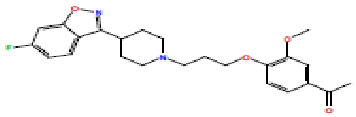<br>DB04946	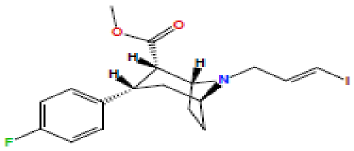<br>DB04947
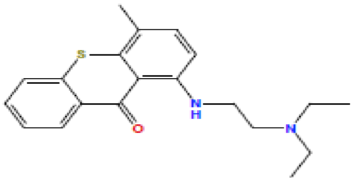<br>DB04967	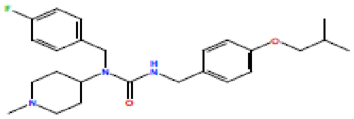<br>DB05316	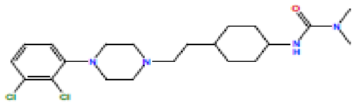<br>DB06016	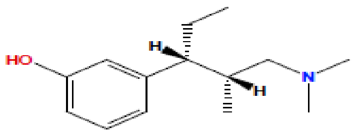<br>DB06204

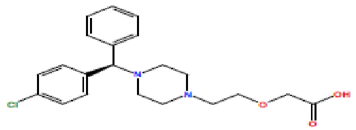<br>DB06282	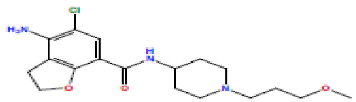<br>DB06480	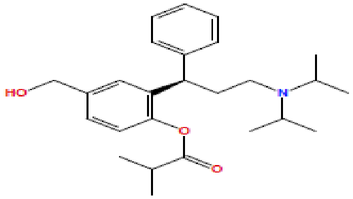<br>DB06702	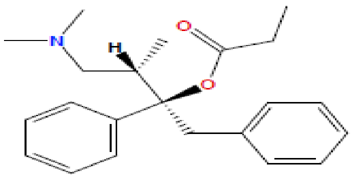<br>DB06793
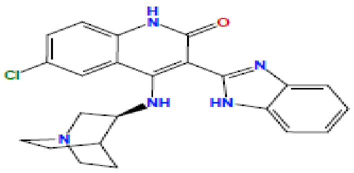<br>DB06852	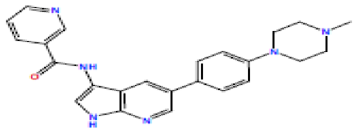<br>DB06876	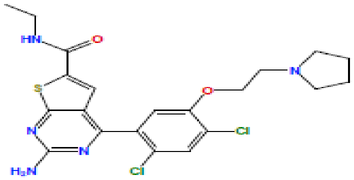<br>DB06969	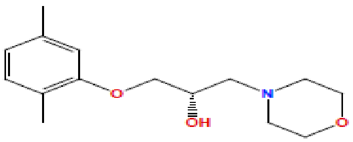<br>DB07573
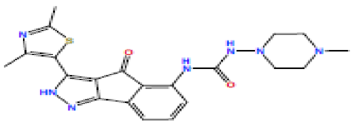<br>DB07622	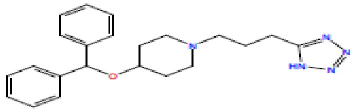<br>DB07917	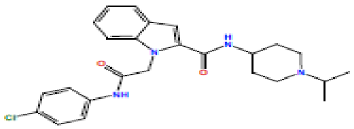<br>DB07974	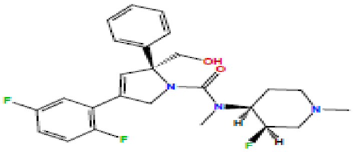<br>DB08037
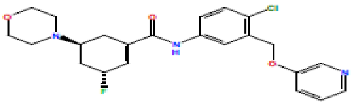<br>DB08068	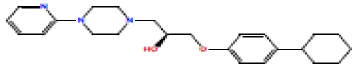<br>DB08543	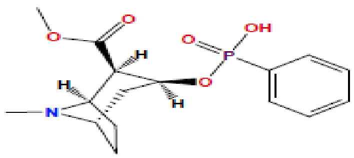<br>DB08618	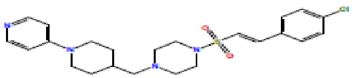<br>DB08746

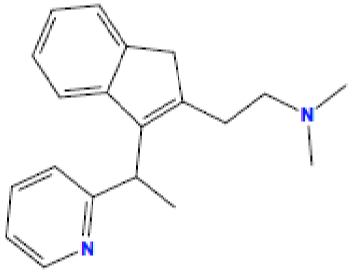 DB08801	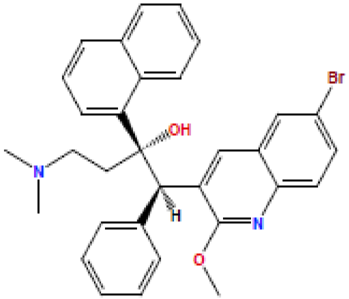 DB08903	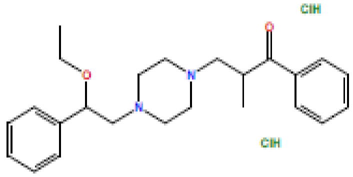 DB08990	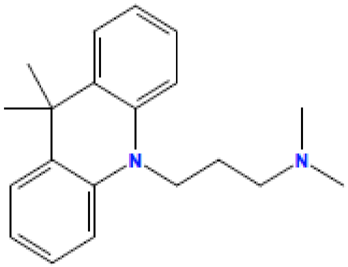 DB08996
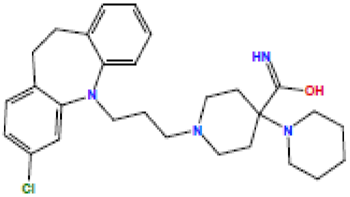 DB09003	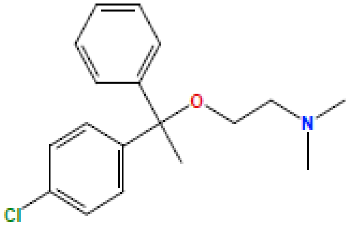 DB09007	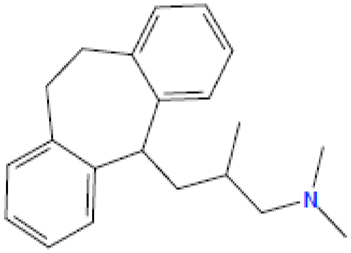 DB09016	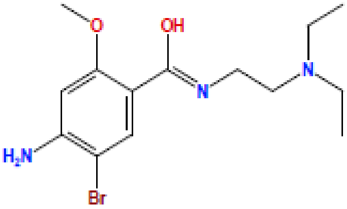 DB09018

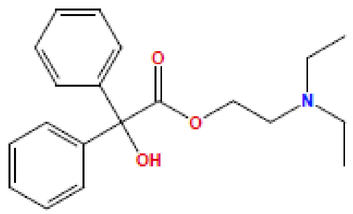

DB09023

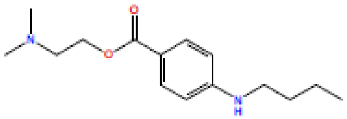

DB09085

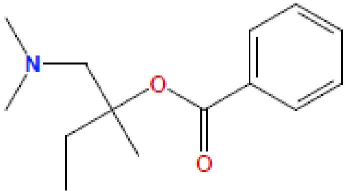

DB09088

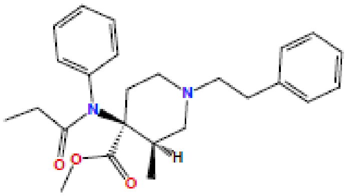

DB09174

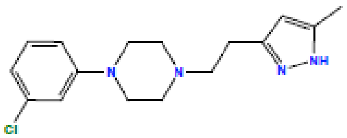

DB09197

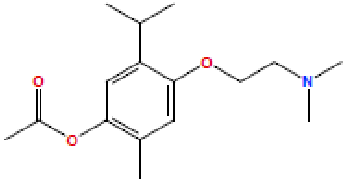

DB09205

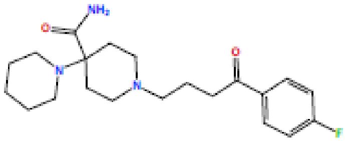

DB09286

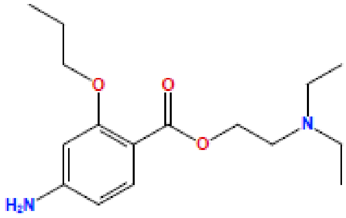

DB09342

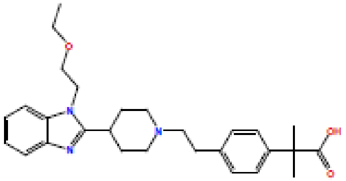

DB11591

C.

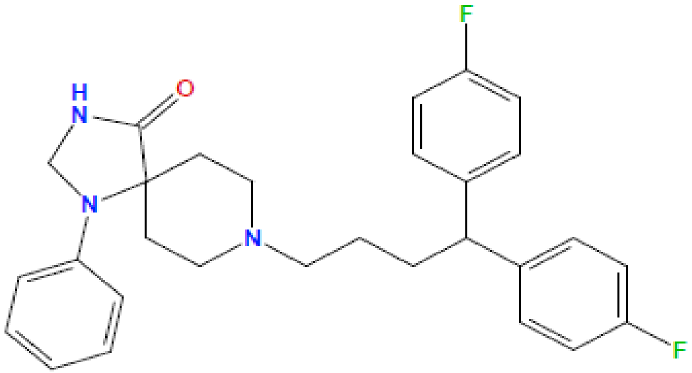 DB04842	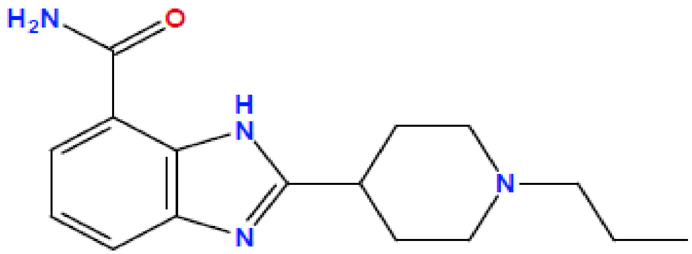 DB07330

D.

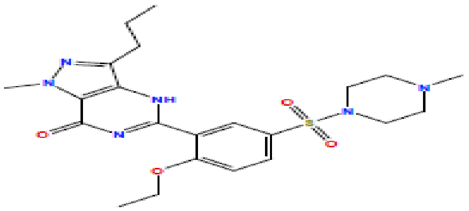 DB00203	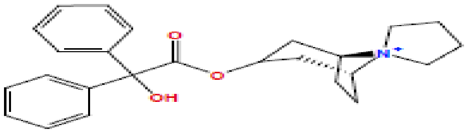 DB00209	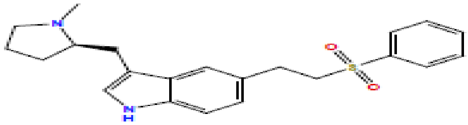 DB00216
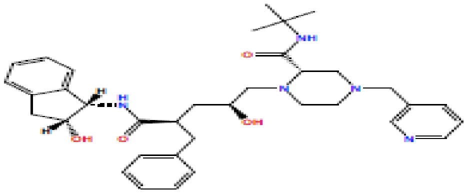 DB00224	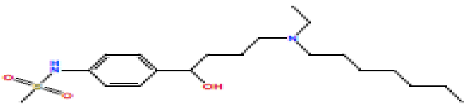 DB00308	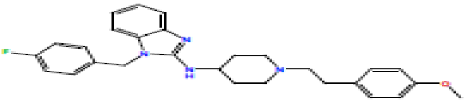 DB00637
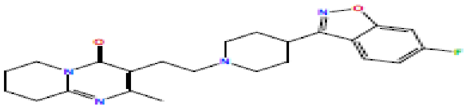 DB00734	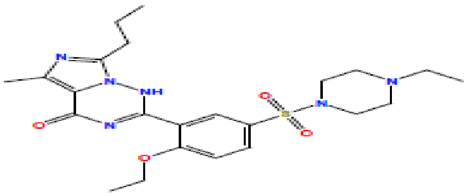 DB00862	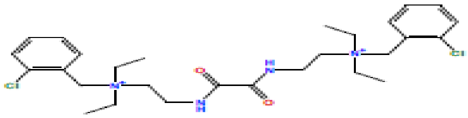 DB01122
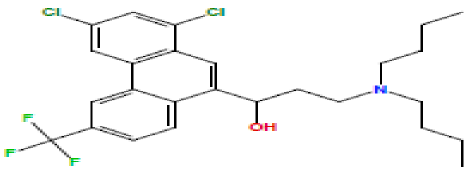 DB01218	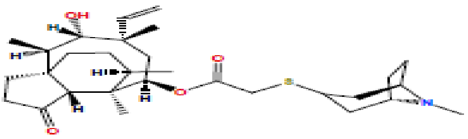 DB01256	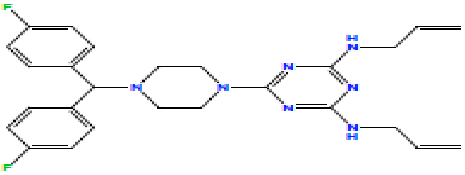 DB01430

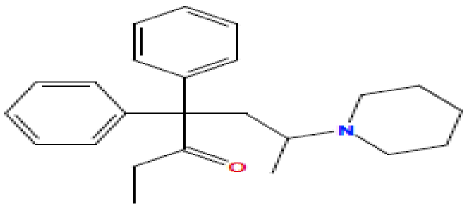 DB01491	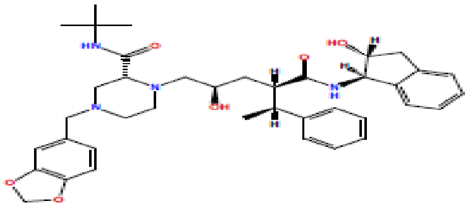 DB01721	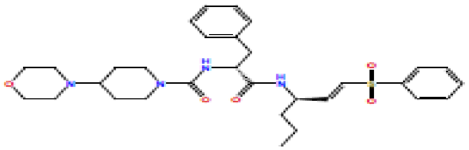 DB02243
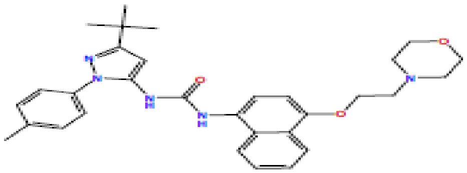 DB03044	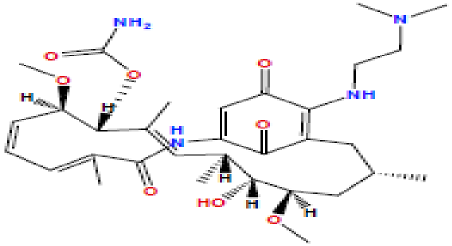 DB03080	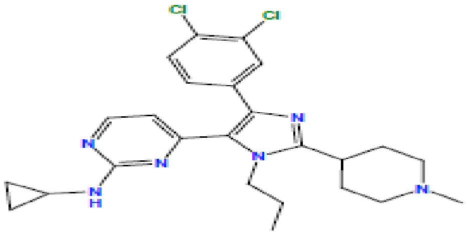 DB03084
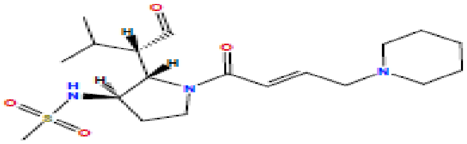 DB03890	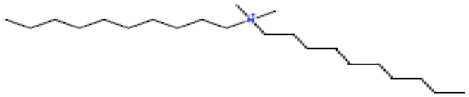 DB04221	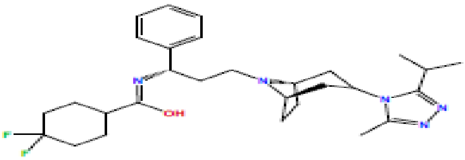 DB04835
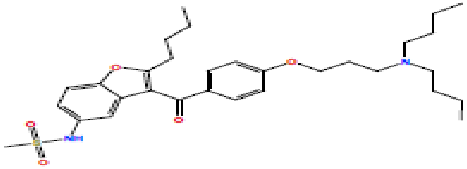 DB04855	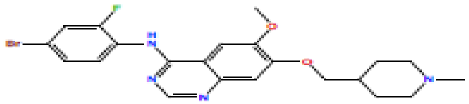 DB05294	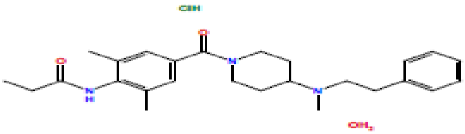 DB05461

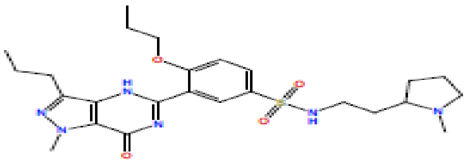 DB06267	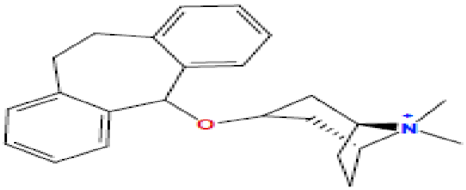 DB07494	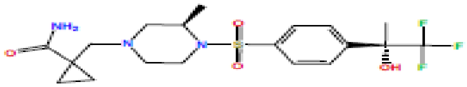 DB07624
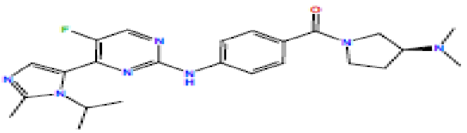 DB07936	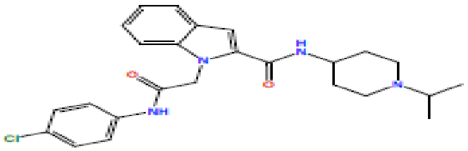 DB07974	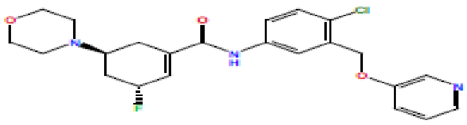 DB08068
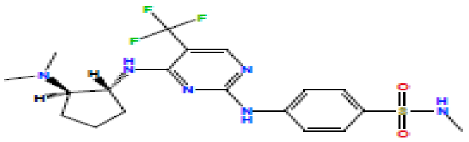 DB08341	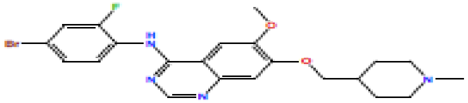 DB08764	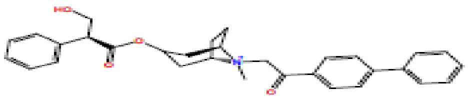 DB08978
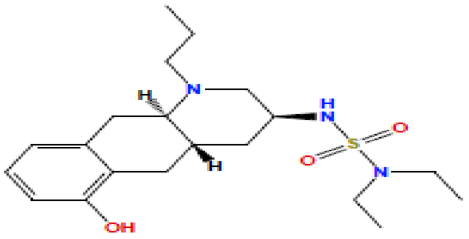 DB09097	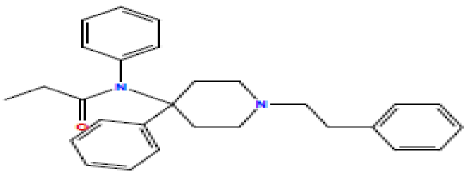 DB09168	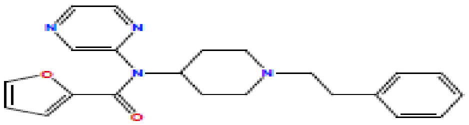 DB09175

The chemical structures of drug candidates were identified in our study. **A.** Cannabinoid 2 receptor agonists (82 drugs); **B.** Histamine 3 receptor antagonists (109 drugs); **C.** Histamine 1 and 4 receptor antagonists (two drugs); **D.** Dopamine 3 receptor antagonists (36 drugs). The figures were generated by the Molecular Operating Environment (MOE), Chemical Computing Group Inc. Montreal, QC, Canada.

Figure S2. Tanimoto similarity Heatmap.

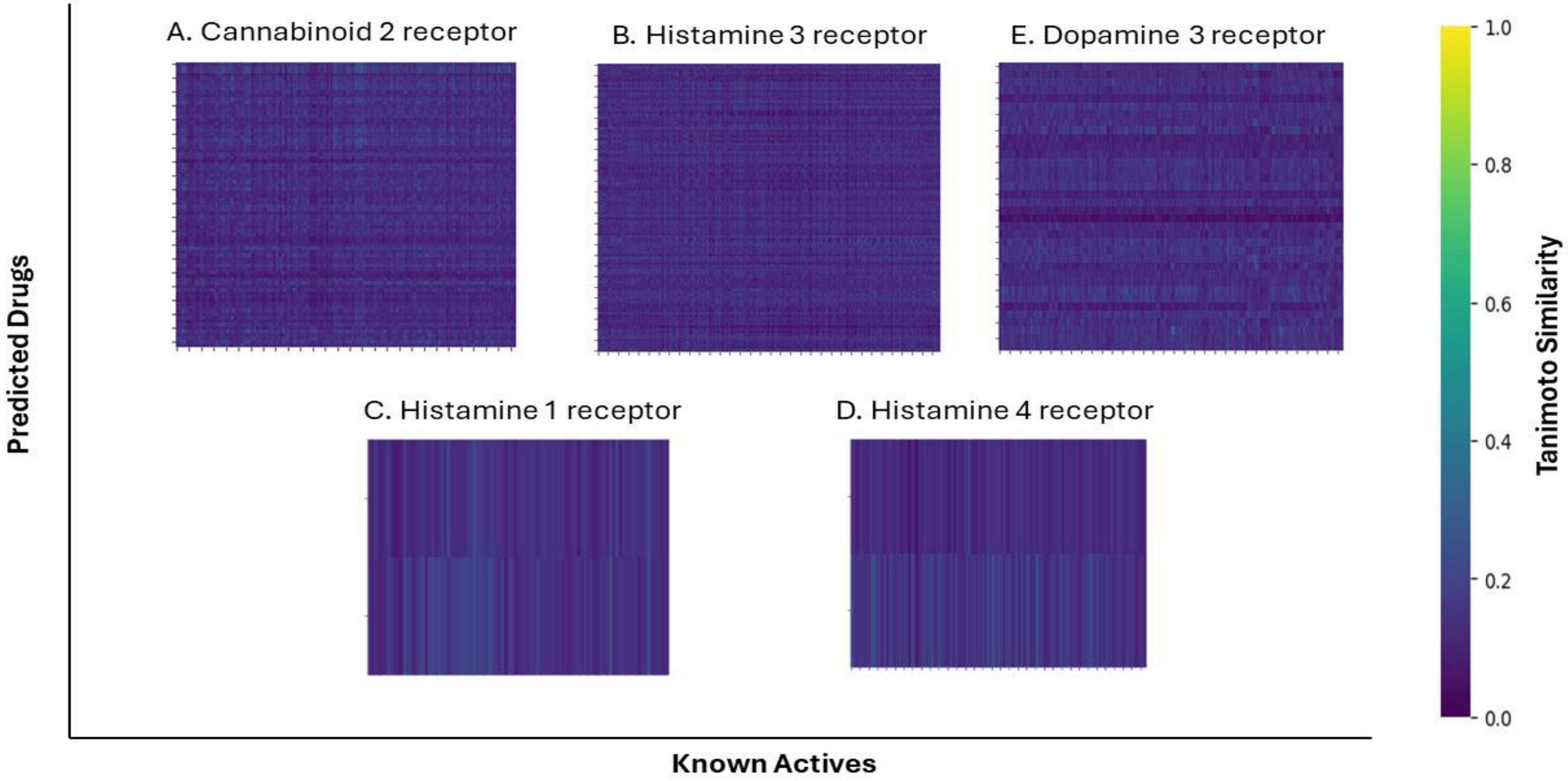

Heat map of molecular similarity plotted by the Tanimoto similarity index (0-1) using ECFP4 fingerprints (RDKit toolkit v. '2024.03.5') for the different receptors: **A.** Cannabinoid 2 receptor (average Tanimoto= 0.13); **B.** Histamine 3 receptor (average Tanimoto= 0.13); **C.** Histamine 1 receptor (average Tanimoto= 0.14); **D.** Histamine 4 receptor (average Tanimoto= 0.14); **E.** Dopamine 3 receptor (average Tanimoto= 0.13). The x-axis and y-axis represent the set of known actives used to build the different models and the predicted drugs to be active on that receptor, respectively.

Figure S3. The redocked ligands aligned to the native pose in each structure.

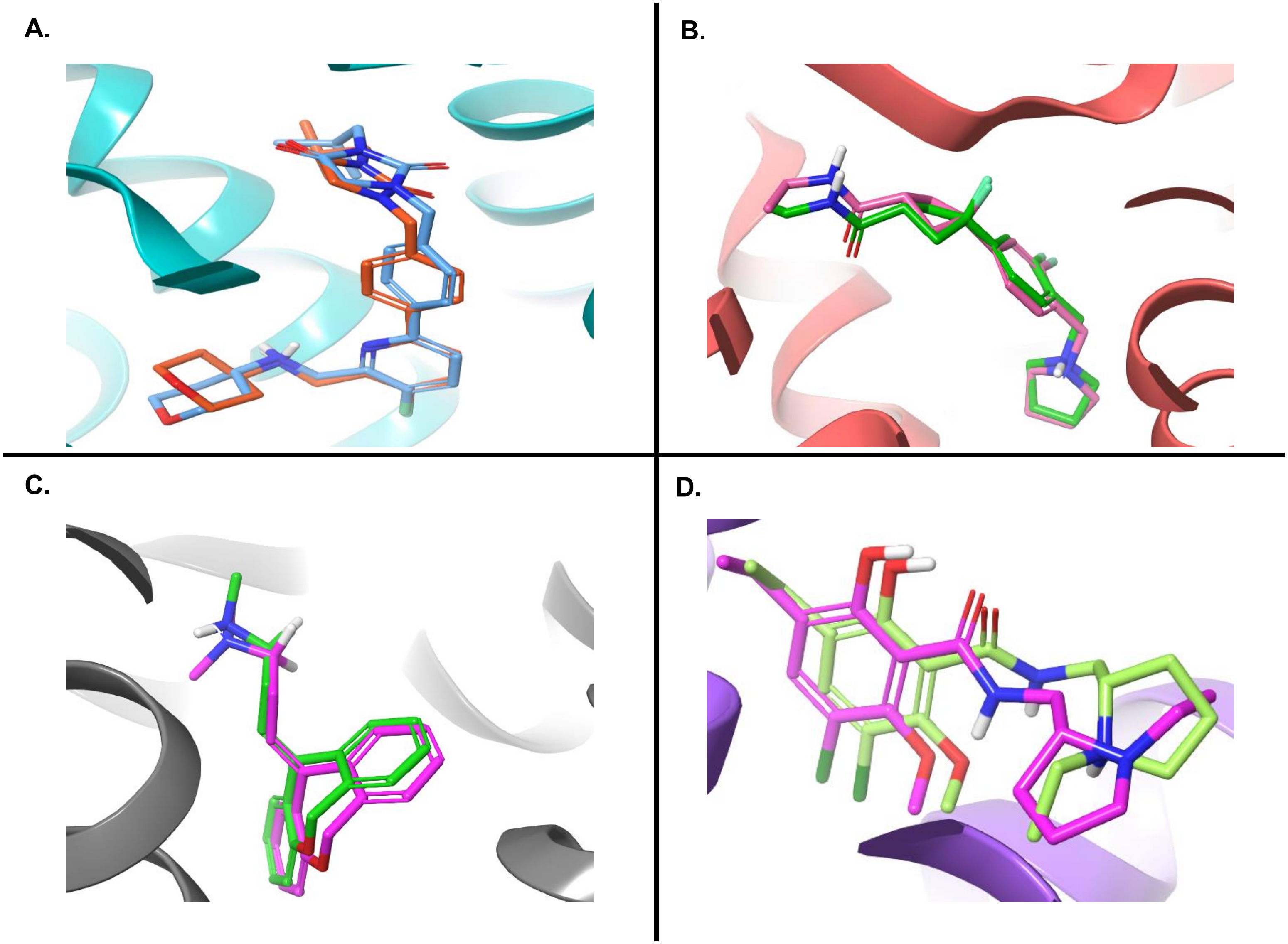

3D representation of the redocked ligands (best pose) aligned on the original (native) pose from the PDB structure. **A.** Cannabinoid 2 receptor (PDB ID: 8GUT, teal) with the native agonist (LEI-102) as orange sticks and the redocked pose as azure sticks (RMSD=2.6 Å); **B.** Histamine 3 receptor (PDB ID: 7F61, red) with the native antagonist (PF-03654746) as green sticks and the redocked pose as pink sticks (RMSD=2.8 Å); **C.** Histamine 1 receptor (PDB ID: 3RZE, gray) with Doxepin (H1R antagonist) as green and pink sticks for the native and redocked poses, respectively (RMSD=2.5 Å); **D.** Dopamine 3 receptor (PDB ID: 3PBL, violet) with the native agonist (LEI-102) as lime sticks and the redocked pose as pink sticks (RMSD=3.8 Å). The figures were generated by Maestro (version 12.9.137, Schrödinger, LLC, New York, NY, 2021).

Figure S4. 2D- representation of Ligand-Protein Interactions in redocking.

A.

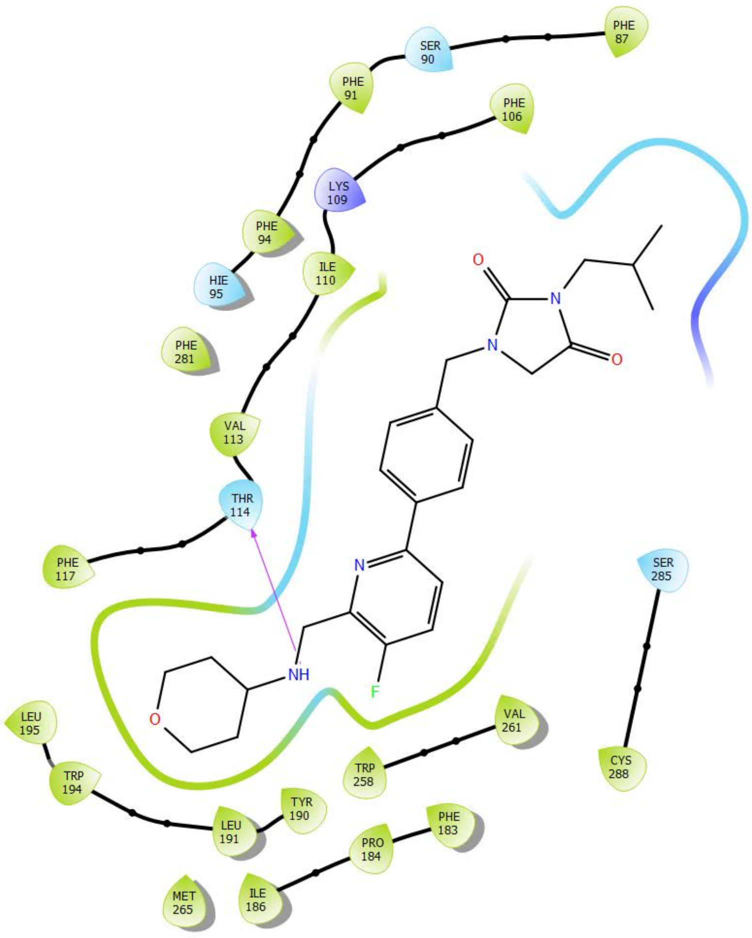

B.

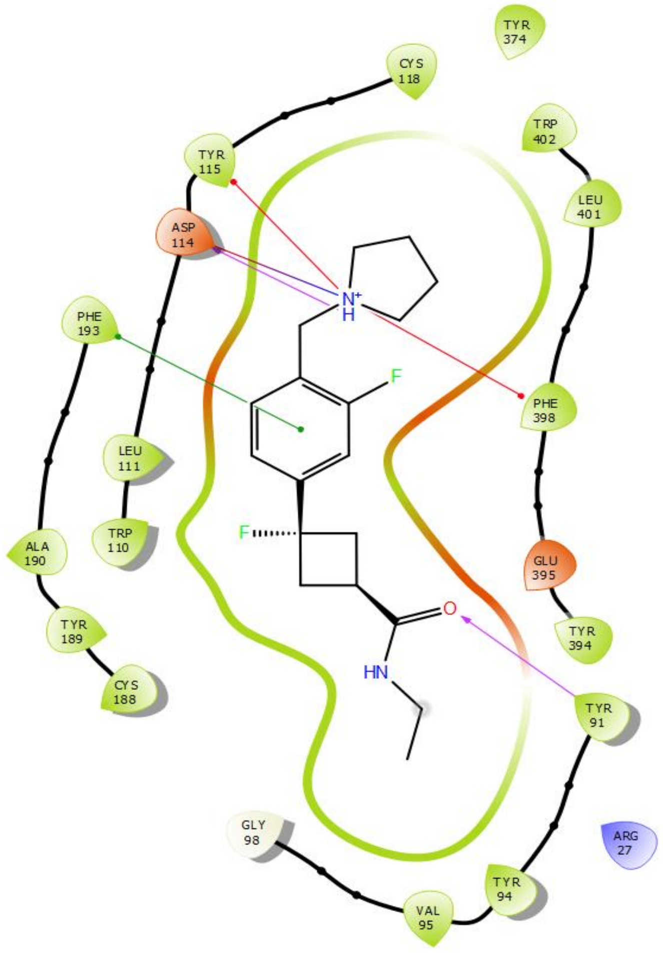

C. **D.**

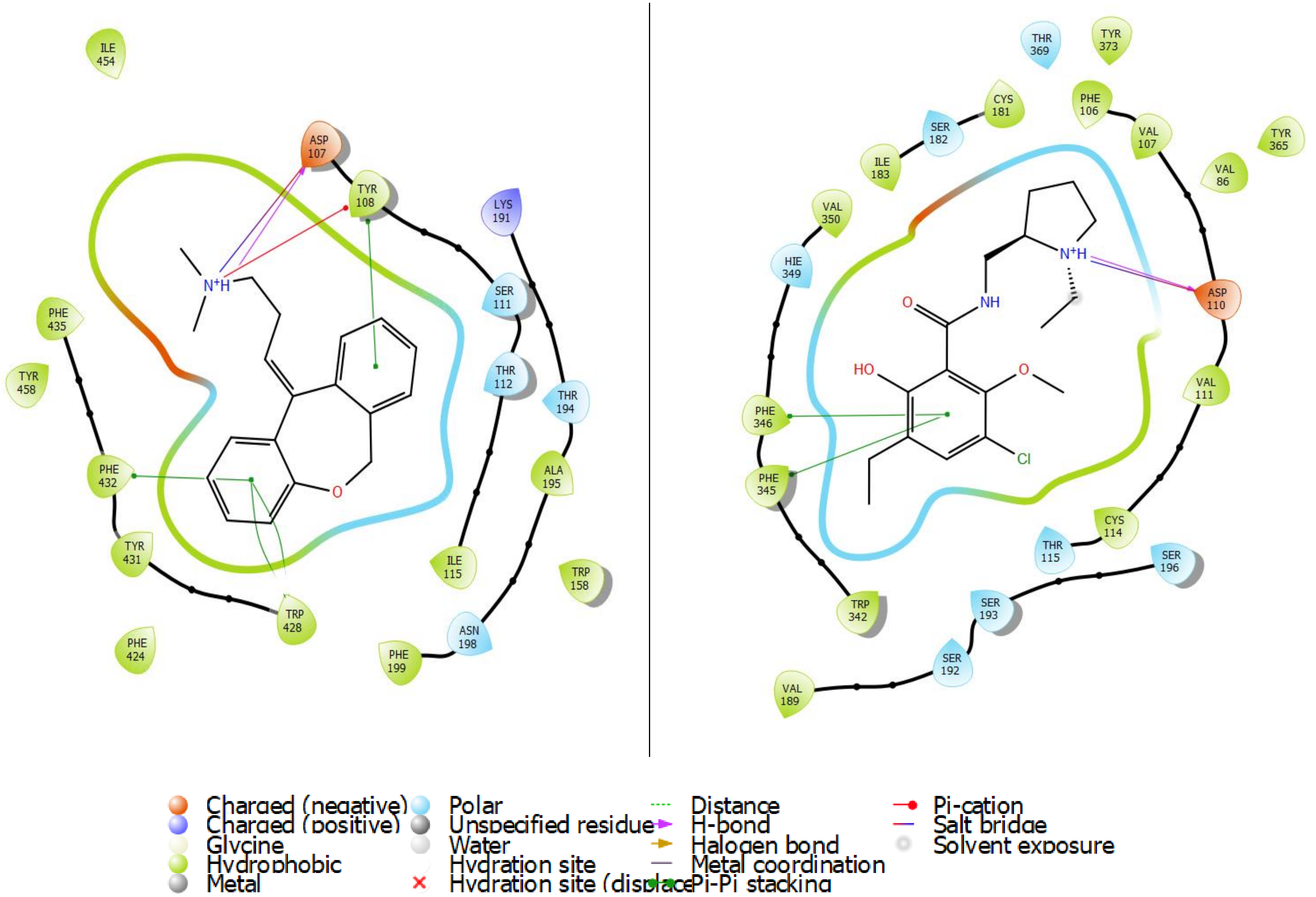
ILE 454
ASP 107
TYR 108
LYS 191
N+H
PHE 435
SER 111
TYR 458
THR 112
THR 194
PHE 432
O
ALA 195
TYR 431
ILE 115
TRP 158
TRP 428
PHE 424
ASN 198
PHE 199
THR 369
TYR 373
CYS 181
PHE 106
SER 182
VAL 107
TYR 365
ILE 183
VAL 86
VAL 350
N+H
HIE 349
NH
O
ASP 110
HO
O
VAL 111
PHE 346
Cl
PHE 345
CYS 114
THR 115
SER 196
TRP 342
SER 193
SER 192
VAL 189
Charged (negative)
Charged (positive)
Glycine
Hydrophobic
Metal
Polar
Unspecified residue
Water
Hydration site
Hydration site (displaced)
Distance
H-bond
Halogen bond
Metal coordination
Pi-Pi stacking
Pi-cation
Salt bridge
Solvent exposure

2D representation of the interactions between the redocked ligands (best pose) and the different receptors used in this study: **A.** LEI-102 (agonist) with the cannabinoid 2 receptor (PDB ID: 8GUT); **B.** PF-03654746 (antagonist) with the histamine 3 receptor (PDB ID: 7F61); **C.** Doxepin (antagonist) with the histamine 1 receptor (PDB ID: 3RZE); **D.** Eticlopride (antagonist) with the dopamine 3 receptor (PDB ID: 3PBL). The legend outlines the different interaction types. The figures were generated by Maestro (version 12.9.137, Schrödinger, LLC, New York, NY, 2021).
